# Structural dynamic studies on identification of EGCG analogues for the inhibition of Human Papillomavirus E7

**DOI:** 10.1038/s41598-020-65446-7

**Published:** 2020-05-26

**Authors:** Murali Aarthy, Umesh Panwar, Sanjeev Kumar Singh

**Affiliations:** Computer Aided Drug Design and Molecular Modeling Lab, Department of Bioinformatics, Alagappa University, Karaikudi, 630004 India

**Keywords:** High-throughput screening, Target validation

## Abstract

High risk human papillomaviruses are highly associated with the cervical carcinoma and the other genital tumors. Development of cervical cancer passes through the multistep process initiated from benign cyst to increasingly severe premalignant dysplastic lesions in an epithelium. Replication of this virus occurs in the fatal differentiating epithelium and involves in the activation of cellular DNA replication proteins. The oncoprotein E7 of human papillomavirus expressed in the lower epithelial layers constrains the cells into S-phase constructing an environment favorable for genome replication and cell proliferation. To date, no suitable drug molecules exist to treat HPV infection whereas anticipation of novel anti-HPV chemotherapies with distinctive mode of actions and identification of potential drugs are crucial to a greater extent. Hence, our present study focused on identification of compounds analogue to EGCG, a green tea molecule which is considered to be safe to use for mammalian systems towards treatment of cancer. A three dimensional similarity search on the small molecule library from natural product database using EGCG identified 11 potential small molecules based on their structural similarity. The docking strategies were implemented with acquired small molecules and identification of the key interactions between protein and compounds were carried out through binding free energy calculations. The conformational changes between the apoprotein and complexes were analyzed through simulation performed thrice demonstrating the dynamical and structural effects of the protein induced by the compounds signifying the domination. The analysis of the conformational stability provoked us to describe the features of the best identified small molecules through electronic structure calculations. Overall, our study provides the basis for structural insights of the identified potential identified small molecules and EGCG. Hence, the identified analogue of EGCG can be potent inhibitors against the HPV 16 E7 oncoprotein.

## Introduction

Cervical cancer is represented as the second most common cancer of women worldwide and is the most common sexually transmitted viral infection^1,2^. A total of 291 million women worldwide are diagnosed as the carriers of HPV which in turn causes cervical cancer^3,4^. The fundamental cause of cervical cancer is the determined infection through the Human Papillomavirus (HPV) and other factors responsible for the progression of the cervical cancer is the age during the first intercourse, several sexual partners, smoking and low socioeconomic status^5,6^. The HPV belongs to the family of Papillomavirus which is heterogeneous and highly species specific. It is circular, double stranded and non-enveloped DNA virus of 8kb^7^ and belongs to the varied group constituting more than 130 genotypes. This virus infects and transmits in several animal species as well as humans^8^. The diversed genotypes of HPV have been classified into low and high risk based on the efficiency to induce transformation or the malignancy. The HPVs type 16, 18, 31, 33, 35, 39, 45, 51, 52, 56, 58, 59, 68, 73 and 82 are considered to be the high risk whereas, the HPVs type 6, 11, 40, 42, 43, 44, 53, 54, 61, 72 and 81 are considered to be the low risk. Also, HPV types 31, 33, 35, 51 and 52 are considered to be the intermediate risk due to the existence in mild as well as in severe lesions^7,9^. The cell cycle of HPV induces propagation of the basal and parabasal cells that leads to the epithelial hyperplasia or papillomatosis with varied extension.

The HPV genome encodes for the several proteins namely E1, E2, L1, L2, E4, E5, E6 and E7. The E1 and E2 protein plays a major role in the replication whereas L1 and L2 protein are involved in the viral genome packaging. In specific, the oncogenic property of HPV is due to the activity of E5, E6 and E7 viral proteins found within the genome. The early ORFs of HPV encodes for three oncoproteins namely E5, E6 and E7^10–12^. A total of five genera for human papillomavirus has been classified namely Alpha, Beta, Gamma, Mu and Nu. The highlights of identification of these genera states that E5 has not been encoded by all genera and only alpha HPVs encode E5 and gets express^10^. HPV E7 oncoprotein plays towards the induction and progression related to cancers as well as the oncogene expression in primary keratinocytes. These expression causes cellular immortalization which exhibits numerous hall marks of premalignant lesions. The cell-cell fusion along with the formation of the tetraploid cell and failure of cytokinesis is induced by the protein E5 of HPV^10^. Also, the malignant transformation of the host cell leading to the tumor formation is mediated through the oncoproteins E6 and E7^13^. These oncoproteins plays foremost part in proliferation of cells and inability to repair the damage in the genetic material which leads to the aneuploidies and mutations developing cancer^5^. It was found that the oncogenic HPVs contribute to phenotypic alterations of cancer like continuous proliferative signalling, avoiding growth suppressors, triggering tissue invasion and metastasis, supporting immortality, inducing angiogenesis and cell death resistance^14^.

The oncoproteins E5, E6 and E7 are comparatively small proteins with an approximate length of 85, 150 and 98 amino acids respectively. These proteins lack enzymatic activities, but influence the host cell by interacting with the cellular proteins. Although E6 and E7 confer crucial transforming activities of human papillomavirus, E5 boosts the function of them which contributes towards the tumor progression^15^. The nature of E5 proteins are hydrophobic and exhibits transforming activity which induces malignant phenotype for the cooperation with E6 and E7. But the E5 protein does not expose any role in the primary conservation of the development in HPV genome or proliferation of the undifferentiated keratinocytes, instead it plays a definite role in the dependent stages of differentiation^16^. E5 protein is considered to be weak but the effect is observed to preeminent when it is cooperated with other viral oncoproteins namely E6 & E7^17,18^. White *et al*., states that numerous cellular proteins have been identified to interact with E5, E6 or E7 and these interactions are believed to possess importance in biological activity of the protein towards cell transformation and fudging of immune response. The domain of the HPV-16 E5 and HPV-31 E5 interacts directly with the heavy chain component of MHC1 through leucine pairs in the region. The ability of E6 and E7 oncoproteins inactivates the classic tumor suppressor p53 and pRb (retinoblastoma) respectively which in turn interrupts the multiple cellular pathways^13,19,20^. The oncoprotein E6 attaches to the cellular ubiquitin ligase E6AP (E6-associated protein) that leads to the conformational shift of E6 and allows the trimetric E6/E6AP/p53 complex and this complex results in the proteolytic degradation of p53^21,22^. Similarly, the E7 protein binds to pRb and degrades the suppressor function for uncontrolled activation of E2F transcription factor that kindles the expression of genes involving in the S phase of the cell cycle^23^. It also interacts with the other cellular targets namely p16^INK4a^, Ki-67 and certain specific inhibitors of cyclin-dependent kinases like p21 and p27. E7 plays a major role in convincing the differentiated cells to enter into the cell cycle^5,24^.

E7 protein does not show any sequence similarities to cellular proteins but then certain E7 motifs particularly the LXCXE sequence is found^25^. The amino terminus of E7 protein shares the functional and sequence features with the adenovirus E1A and SV40 antigen^26^. The E7 oncoprotein forms tetramer and higher order oligomers which may possess chaperone activity^15^. The C-terminal domain of HPV type 1 and 45 that possess the characteristics of zinc binding is available as a three dimensional structure and this encompasses amino acids 44–93^27–29^. It is also observed that the residues 1–20 are stated as the conserved domain 1 (CD1) which possess the acute locus to induce S-phase progression and cellular transformation that binds with the cellular targets like UBR4/p600, SKp2 and p300. Some of the binding partners which interact with this domain are p300/CBP associated factor. The residues 20–38 has been revealed to be conserved domain 2 which comprehends the site of CKII phosphorylation and the motif LXCXE domain which is involved in the binding of the tumor suppressor Rb1. Also it is very efficient for the retraction of the anti-proliferative signals and the oncogenic transformation. The CD2 is pivotal for the transforming capacity of E7 and the aptitude to initiate progression of S-phase. The conserved domain 1 and 2 exist at the N-terminus of the protein in disordered arrangement whereas the CD3 region is signified to be ordered in the C-terminal comprising of β1β2α1β3α2 fold^30–35^.

E7 forms a stable structure although the amino terminal CD1/CD2 sequences represent the intrinsically disordered domain^27^. CD3 constitutes the region containing CXXC motifs within the residues 38–98 which is responsible for the zinc binding and this motif is involved in the dimerization of the protein^31,36^. Also, this domain involves in the interaction with various cellular proteins namely p21 and p27 CDK inhibitors and interacts with the Mi2β component of the histone deacetylase complex and TBP^36^. It also involves in the degradation of pRb^26^.In general, it is noticeably implicit that these domains are essential for the abrogation of epithelial cell quiescence and they also contribute to the cellular transformation^31,37,38^.

Despite the potential therapeutic relevance, numerous chemotherapeutical agents are available for use in the therapy against cervical cancers. But they are known to cause cytotoxicity to normal cells. Hence, the significance to develop drugs that specifically kills the cancer cells appears as a prerequisite^39^. Generous reports of epidemiological and experimental studies portrayed the relationship between tea and cancer prevention. Ahne *et al*., have confirmed that the green tea component (-)-Epigallocatechin-3-gallate possess the anti-cancer effects and suppress the growth of cancer cells from various human origins inclusive of HPV infected cervical cancer cell lines^39–41^. The effect of EGCG, green tea component shows inhibition on carcinogenesis which is induced by the wide variety of carcinogens in rodent cancer models^42^. EGCG is represented as the major bioactive polyphenol present in the green tea and possess anti-angiogenic, anti-proliferative, anti-metastatic, proapoptotic and cell cycle perturbation in various *in vitro* and *in vivo* tumor models^43^.

The effect of EGCG on signalling of EGFR in various cervical cells demonstrates that it inhibits EGFR which is an initial kinase in the EGF signalling cascade. This inhibition through EGCG is associated with the phosphorylation reduction level that leads to G1 arrest and apoptosis increase^44^. Nair *et al*., states that the over expression of aromatase in HPV positive cervical cancer cells resulted in increased expression and activity of Estrogen Receptor, higher expression of E6/E7 and increased cell proliferation^45^. It is evident with several reports that the expression of E6 and E7 of HPV is affected by the EGCG. It suppresses the protein expression of ERα and aromatase which indirectly confines the expression of E6/E7 and in turn inhibits the cervical cancer cell growth and apoptosis^46^. Hence, our study mainly focuses on the identification of compounds analogue to (-)-Epigallocatechin-3-gallate through the computational approach which helps to streamline the discovery of potent inhibitors. Herein, we have incorporated the shape based screening approach to identify the small molecules equivalent to EGCG. Further, the screened molecules were taken forward for Glide XP docking on HPV E5, E6 and E7 in order to understand the role of analogues compound in the inhibition of HPV oncoproteins since, all three oncoproteins depicts significant role in the development of positive cancer cell growth. Also, we have concentrated on the aromatase and Estrogen Receptor α which helps in the expression of E6/E7 oncoproteins. Further, induced fit docking strategy has been carried out along with the post docking and post dynamics binding free energy calculation on HPV 16 E7. In order to gain overall insights into the binding of the compounds to the active site of HPV 16 E7 and their stability, we attempted molecular dynamics simulation studies. This also helped in the assessment of the conformational changes and the dynamic behaviour of the protein. We have also performed an additional simulation of twice for the complexes and apoprotein for the period of same 100 ns to obtain statistically valid results on conformational stability. The best identified small molecule compounds finalized from the conformational stability check has been carried forward for the analysis of the molecular features through the electronic structure calculation and the drug like properties of the identified compounds is analyzed by the ADME/T prediction. This study provides a gateway for the identification of novel and potent inhibitors analogue to the molecule EGCG which could be a potent anti-HPV agents.

## Materials and Methods

### Ligand preparation

Green tea component, (-)-Epigallocatechin-3-gallate (EGCG) inhibits the development of cervical cancer through apoptosis generation, cell cycle arrest and regulation of gene expression. The two dimensional chemical structure of EGCG was retrieved from the PubChem database and the structure were prepared through LigPrep application implemented in Schrödinger^47,48^. The hydrogen atoms were added to the ligand molecules and the bond orders were fixed whereas the ionization states for the ligand structure were specified at certain pH of 7.0 using Epik. The force field OPLS-2005 was used and a maximum of 32 possible conformations were generated stating that the ligand molecule were further ready to be used in the theoretical calculation^49^.

### Shape based screening

The significant technique which utilizes the electrostatic potential and shape of a compound for identification of compounds with similar structural arrangement is the phase shape based screening^50^. The structural arrangement and properties of the template structure will be used for the identification of similar molecules in the database^51^. The shape based screening application employed in Maestro 10.4 screens the prepared ZINC and NCI small molecule databases against the template structure. Based on the scores obtained from the similarity index of the template and the identified small molecules received, the molecules with best scores were retrieved and further utilized for docking strategies. The scores provided based on the similarity were calculated using the pharmacophore feature types in order to calculate the overlapping of volume which is shown to outperform the use of atom types^52^. The maximum volume observed during the overlap of compounds was used to establish the best alignments since the similarity were counted only for the pharmacophoric features of similar type^53^. The atoms treated by the shape search are analogous and it incorporates the information of the available atom types of template. Investigation on the varied alignments rather than the characteristic search that equalize the hypothesis is executed by the shape query. Evaluation on the similarity of the shape and scores of the volume has been carried out by the alignment algorithm^54^.

### Molecular modeling environment setup

The high quality three dimensional homology model of HPV type 16 E7 protein is considered to be the primary source, which is built using the MODELLER version 9.16^55^ as described in our earlier work^8^. Also, the three dimensional structure of HPV E5 has been developed with the template 4L9P using Modeller and has generated the low energy conformation through dynamic simulation studies and the results were represented in Supplementary Figure 1 whereas the crystal structure of E6 has been retrieved from PDB with the ID 4XR8. It was clearly reported that the HPV positive cervical cancer cells shows aromatase to be over expressed resulting in higher expression of E6/E7, increased activity of ERα and increase in cell proliferation. Hence, with this as a verge, we studied the inhibition of aromatase (PDB ID: 3EQM) and ERα (PDB ID: 1XQC) that corresponds to the expression of E6/E7 which in turn helps in the inhibition of E6/E7. The proteins downloaded and modeled was not ready to be used in the docking protocol and other molecular modeling studies, hence the proteins were prepared through the application of Maestro 10.4^56^. This module helps in assigning the bond order that gives the missing information and further the hydrogen atoms were also added. Generation of tautomeric states and protonation of His were performed to optimize the rotating hydroxyl, thiol hydrogens and hydrogen bonding network. Minimization of the modeled structure has been carried out with the help of OPLS-AA (Optimized potentials for Liquid simulation on All Atoms) force field till the rmsd of non-hydrogen atoms reaches 0.30 Å^57^. The structure of E5, E6, Aromatase and ERα has been represented in the Supplementary Figure 2.

### Site prediction based grid generation

The vdW charges on the proteins E5, E6 & E7 surface helps in predicting the druggability sites through the clustering of favourable regions with the SiteMap available in Schrödinger ^58,59^. This module includes the OPLS-2005 force field parameters that plays significant role in estimating the interaction energies of probe positioned at three dimensional grids that encompass the entire protein^60^. The druggability site obtained with the best scored site was revealed to be the source and the generation of the grid which was performed by picking the white coloured spheres available in the predicted site of the receptor^61^. The van der Waals radius of scaling factor with 1.0 and partial charge cut off of 0.25 helps in the determination of the ligand’s position by manually picking the white spheres. The position of the grid box for all the proteins was fixed on the XYZ axes and has been represented in Table 1 in the supplementary information whereas the grid position for E7 has been described from our earlier work^8,62^.

### Receptor information based grid generation

In order to generate the grid for the receptor aromatase and ERα, the ligand information available on the crystal structure 3EQM and 1XQC were utilized by picking the component manually which indicates the centroid of the binding site in the receptor containing the residues responsible for the ligand to interact. Selection of sites in the receptor accountable for active involvement of binding with the ligand for the constraint of hbonds is represented as flexible residues. The position of the spherical region that should be occupied by a particular ligand during docking was set on xyz axes with co-ordinates of 85.65, 54.117 and 45.84 respectively for aromatase and 14.12, 3.65 and 36.48 for ERα respectively^63^.

### Extra precision GLIDE docking

The small molecules identified from the databases with the template structure as EGCG were docked into the binding site of HPV 16 E5, E6 & E7 protein along with the aromatase and ERα using Glide (Grid based Ligand docking with Energetics) available in Schrödinger^64,65^. Filtration based on the hierarchical series in order to search the possible locations of ligand in the active site region of the receptor is implemented with glide approach. The grid based method uses the initial filters which helps in examining the receptor ligand interactions balance and testing the spatial fit of the ligand to the definite active site. Various conformations were generated internally by this approach and were conceded through the series of filters during the maintenance of sampling flexible ligand. The ligand’s were positioned and permitted to move around three angles during the preliminary stage followed by the removal of dissimilar binding modes available with the docking scores of the entities and geometrical filter. Finally, evaluation and refinement of the docking which includes the torsional and rigid body movements of the ligand were performed with the help of OPLS-AA force field^66^. The scaling factor of 0.8 Å and a partial charge cut off of 0.15 were used for van der Waals scaling of ligand. The docking process involves the prediction of conformation and orientation of the ligand encompassing the binding site of the target molecule. The ligands with the best scores and interactions were selected for further analysis.

### Induced fit docking

The energy calculation of the ligand has been executed with the aid of diverse charges and follows three consecutive steps in induced fit docking approach. During the initial stage, docking of ligand to the rigid receptor model is executed with the scaling of van der Waals (vdW) radii. Also, the docking approach accounts for minimum backbone relaxations along with the side chain conformational changes in the receptor which will be validated with large set of pharmaceutical example. Availability of non-polar atoms in receptor as well as the ligand were employed through the van der Waals scaling with the measure of 0.5 Å. The challenges that occur during the reorganization of the receptor protein upon binding with the small molecule are decreased only with the employment of induced fit docking strategy. The removal of bad steric contacts and the constrained energy minimization was carried out on the protein structure with the OPLS-2005 force field and the implicit solvation model until the default criteria were met^66^. During the implementation of standard precision mode in the initial docking phase of induced fit docking, 20 ligand poses and protein ligand contact were retained for the structural refinements. The backbone and the side chain refinement gets assigned by the docked conformers in the earlier step. The receptor structures were obtained with the minimum energy of 30 kcal/mol and were delivered through the final stage of glide docking and scoring. Later, the ligand is redocked into the refined low-energy receptor structure which is produced in second stage with the help of XP protocol. The top ranked IFD poses were accounted with the protein-ligand interaction energy along with the calculation of total energy of the system^62,64^.

### ADME analysis

The best identified small molecule compounds utilized for the docking strategies were studied for their absorption, distribution, metabolism and excretion (ADME) properties. The ADME properties predicting application of Schrödinger named QikProp version 4.6 was implemented to make accurate predictions on several physically significant ADME property descriptors and pharmaceutically relevant properties of the chosen ligands^41^. This application performs a detailed evaluation of important properties like log P, human oral absorption and Lipinski’s rule of 5 which are considered significant in rational drug design^8^. QikProp uses the BOSS program with OPLS-AA force field to perform Monte Carlo statistical mechanics simulations on organic solutes in a periodic box of explicit water molecules^54^.

### Post docking binding energy calculation

The set of compounds obtained from the screening strategies were carried out for binding free energy calculation using Prime MMGBSA method^65^. It is an efficient method used to refine and rescore the compounds that binds to the target. The complexes were analyzed for the energy through the local optimization feature that helps for the minimization of the docked poses in prime and calculated with the Generalized Born/Surface Area (GB/SA) continuum solvent model and the OPLS force field^52^. The following equations are used for the calculation of the binding free energy.

1 $${\Delta {\rm{G}}}_{{\rm{bind}}}={\Delta }_{{\rm{E}}}+{\Delta {\rm{G}}}_{{\rm{solv}}}+{\Delta {\rm{G}}}_{{\rm{SA}}}$$

2 $${\Delta }_{{\rm{E}}}={{\rm{E}}}_{{\rm{complex}}}-{{\rm{E}}}_{{\rm{protein}}}-{{\rm{E}}}_{{\rm{ligand}}}$$

where, E_complex_, E_protein_ and E_ligand_ are the minimized energies of the protein-inhibitor complex, protein and ligand, respectively.

3 $${\Delta {\rm{G}}}_{{\rm{solv}}}={{\rm{G}}}_{{\rm{solv}}}({\rm{complex}})-{{\rm{G}}}_{{\rm{solv}}}({\rm{protein}})-{{\rm{G}}}_{{\rm{solv}}}({\rm{ligand}})$$

where, ΔG_solv_ is generalized born electrostatic solvation energy of the complex and ΔG_SA_ is a non- polar contribution to the solvation energy due to the surface area.

4 $${\Delta {\rm{G}}}_{{\rm{SA}}}={{\rm{G}}}_{{\rm{SA}}}({\rm{complex}})-{{\rm{G}}}_{{\rm{SA}}}({\rm{protein}})-{{\rm{G}}}_{{\rm{SA}}}({\rm{ligand}})$$

where, ΔG_SA_(complex), G_SA_(protein), and G_SA_(ligand) are the surface area energies for the complex, protein and ligand, respectively. Prime uses a surface generalized Born (SGB) model employing the Gaussian surface instead of a vanderwaals surface for better representation of the solvent-accessible area^8,67^.

### Density functional theory studies

Density functional theory is a quantum mechanical method used widely in the calculation of electronic structure of atoms, molecules and solids which is recently in the increased level of modeling the complex systems that arises in biology & materials sciences. Hence, the larger molecular systems can be studied with the sufficient accuracy which helps in expanding the prediction power inherited in electronic structure theory^68^. The core technology that underlies with DFT is the computational solution of the electronic Schrödinger equation which gives rise to the position of the collection of atomic nuclei and the total number of electrons in the system. This analysis also helps in the calculation of the electronic density, electronic energy and the other properties by means of a defined automated approximation^69^. The molecular electrostatic potential helps in understanding the relative polarity of the molecule whereas the electrophilic and the nucleophilic reactions were interpreted and predicted. The electrostatic potential at the surface of the molecule appears with different colours^70^. In order to analyze the quantum mechanical studies of the compounds, the ligands with the favourable binding poses after interaction studies were subjected to DFT calculation. The analysis were carried out further using the frontier molecular orbitals namely the highest occupied molecular orbitals (HOMO), Lowest unoccupied molecular orbitals (LUMO) and the HOMO-LUMO energy gap. The HOMO energy values depict the ability of the ligands to donate the electrons and the LUMO energy depicts the capability of the ligand to accept electrons^71^. The transition state has been developed due to the interaction between the frontier orbitals of the reactants.

### Molecular dynamics simulation studies

The modeled structure of HPV E7 obtained using MODELLER as described in our earlier work^8^ along with the docked complex of HPV with ZINC14436185, ZINC49069570, ZINC49115270, ZINC14642643 and (-)-Epigallocatechin-3-gallate were carried forward for the molecular dynamics simulation using GROMACS 5.16^66,72,73^. The simulation of the apoprotein along with zinc and protein-ligand systems were performed for a period of 100 ns timescale in order to develop an insight about the atoms present in the protein and its conformational changes in the dynamic environment^74^. Further, it is also very essential to carry out the simulation thrice in order to bring statistically valid output of the molecular dynamics simulation^75,76^. A total of twenty one simulation for a period of 100 ns is carried out including the apoprotein and complexes. PRODRG server and Gromacs utilities have been used for the generation of topologies for the compound obtained from ZINC and NCI database. The structure of the protein complex has been relaxed using the simple point charge (SPC) water model through the elimination of bad contacts and solvating under the periodic boundary conditions. In order to neutralize the net charge of the system, an appropriate number of counter ions like Na+ and Cl- were added and GROMOS96 54a7 force fields were implemented which improves the stability of the secondary structure elements with the assistance of steepest algorithms. The long range electrostatic interactions were calculated with the help of PME (Particle-Mesh Ewald) method^8,77^. The Berendsen thermostat with the coupling time of 0.1 ps were incorporated in order to maintain constant temperature of 300 K in the NVT ensemble and the pressure was maintained with the coupling to the reference pressure of 1 atm. The covalent bond lengths and the geometry of water molecules were constrained with the SETTLE and LINCS algorithms^78^. Hence, simulation has been performed thrice to check the stability of the protein as well the conformational differences. These simulations helps in concluding the stability and depicts the statistical significance in the result obtained^79,80^. The trajectories were analyzed with the GROMACS utilities whereas the quality assurances of all the molecules were obtained and g_hbond were used to cross-check the docking results regarding hydrogen bonding pattern^81^. The secondary structure elements of the apo-protein and the protein complexes were analyzed through the software tool DSSP implemented in GROMACS. This analysis helps to enable a local structural analysis balancing the description of the molecular dynamics studies. The numbers of hydrogen bonds are prominent when the donor-acceptor distance is smaller than 3.9 Å and donor-hydrogen acceptor angle is larger than 90° ^82,83^.

### Post dynamics binding free energy calculation

The method which has been adopted extensively for the estimation of affinity between protein – ligand is the MMPBSA methods which is highly competent and hold high correlation with the experimental values^84^. This method appears to be proficient and responsible to model molecular recognition for receptor-ligand interactions^85^ and employed mostly during the calculation of binding free energy with the trajectory simulations which help in analyzing the binding conformations of the small molecules to the receptor^86^. The binding free energy of the bound ligand – receptor complex in an aqueous solvent can be approximated as follows

5 $${\Delta {\rm{G}}}_{{\rm{bind}},{\rm{aq}}}=\Delta {\rm{H}}-{\rm{T}}\Delta {\rm{S}}\gg {\Delta {\rm{E}}}_{{\rm{MM}}}+{\Delta {\rm{G}}}_{{\rm{bind}},{\rm{solv}}}-{\rm{T}}\Delta {\rm{S}}$$

where ΔE_MM_, ΔG_bind,solv_, and TΔS represent the gas-phase molecular mechanical energy, the solvation free energy change, and the conformational entropy change upon binding, respectively.

6 $${\Delta {\rm{E}}}_{{\rm{MM}}}={\Delta {\rm{E}}}_{{\rm{covalent}}}+{\Delta {\rm{E}}}_{{\rm{elctrostatic}}}+{\Delta {\rm{E}}}_{{\rm{vdW}}}$$

whereas ΔE_MM_ includes three terms that has been calculated using molecular mechanics (MM) namely the covalent energy change (ΔE_covalent_), the electrostatic energy change (ΔE_elctrostatic_) and the van der Waals energy change (ΔE_vdW_).

7 $${\Delta {\rm{E}}}_{{\rm{covalent}}}={\Delta {\rm{E}}}_{{\rm{bond}}}+{\Delta {\rm{E}}}_{{\rm{angle}}}+{\Delta {\rm{E}}}_{{\rm{torsion}}}$$

8 $${\Delta {\rm{G}}}_{{\rm{bind}},{\rm{solv}}}={\Delta {\rm{G}}}_{{\rm{polar}}}+{\Delta {\rm{G}}}_{{\rm{non}}-{\rm{polar}}}$$

ΔE_covalent_ consists of the changes in the terms of bond (ΔE_bond_), the terms of angle (ΔE_angle_), and the torsion (ΔE_torsion_) whereas the solvation free energy change (ΔG_bind,solv_) is usually separated into polar and non-polar contributions namely the ΔG_polar_ and ΔG_non-polar_.

The Molecular Mechanics/Poisson-Boltzmann Surface Area (MM/PBSA) method is widely used method for binding free energy calculation from the snapshots of MD trajectory. The snapshots of the structure is obtained at various time points during the production molecular dynamics simulation of 100 ns and it is used to calculate average values and uncertainties of various quantities of interest^87^.

### Principal component analysis

Principle component analysis facilitates the characterization of folding and unfolding feature of protein along with its dynamic nature of conformation^88^. It also investigates the degrees of freedom of protein which can be reduced. The analysis on the dynamic nature of protein and the correlation between the atomic positional fluctuations derived from molecular dynamics trajectories is considered through the diagnolization of the covariance matrix in PCA. The correlations are expressed as a covariance matrix mentioned below.

9 $$Cij= < \,\delta {x}_{i}\delta {x}_{j}\, > $$

Where, δx_i_ = x_i_ = −<x_i_> and x_i_ represents the coordinates of the Cα at any time and <x_j_> represents the MD coordinates of the same Cα atom i. The variance in the coordinates of Cα atom is represented as δx_j_whereas <δx_i_δx_j_> represents the average covariance between the positions of the Cα atom i and j. The PCA analysis were carried out for the modelled apoprotein and the docked complexes of the compounds ZINC49069570 and EGCG

## Results

### Shape based screening

**Figure 1:**
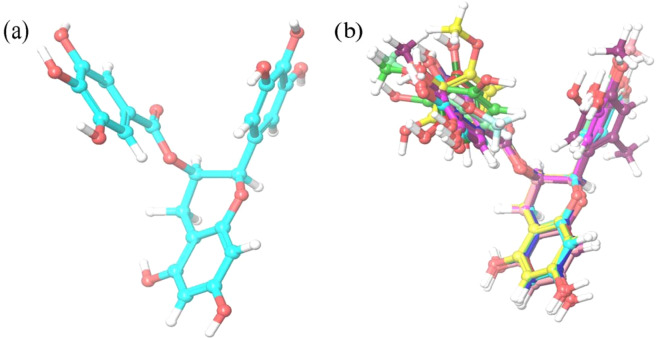
(**a**) Structure of EGCG and (**b**) superimposition of all the retrieved 11 compounds analogue to EGCG.

In the present study, the compound (-)-Epigallocatechin-3-gallate(EGCG), the principal polyphenol component in green tea has been addressed to be a potent chemo preventive agent. This compound exhibits the efficacy of growth inhibition with the estimated IC_50_ values of 51.6 *μ*M for HeLa cell which is used for the cervical cancer studies^36^. The compound EGCG represented in Fig. 1(a) is retrieved from the PubChem database and prepared in the LigPrep module implemented in Maestro 10.4. The conformation of EGCG obtained through LigPrep was used as the template structure for screening from the ZINC and NCI natural product databases. Shape based screening has been considered to be the valuable tool in computer-aided drug design and has successful application like scaffold hopping, bioisostere replacement, virtual library design and flexible ligand superposition. Each conformer from the molecule in database is aligned to the template in diversed approaches and a similarity is computed based on the overlapping hard-sphere volumes.

The conformer and the alignment, yielding the highest similarity for each molecule was identified. The compounds retrieved from the databases were subjected with the phase sim_score and as a result, a total of 11 compounds were retrieved from both the databases with the similarity score of above 0.7 and were listed in the Table 1. Fig. 1(b) represents the superimposed structure of the retrieved compounds and the template structure which shows that the obtained compounds have high similarity with the reference structure.

**Table 1:** Shape Similarity scores and Docking scores of the screened compounds and Epigallocatechingallate.

S.No	Compound Name	Similarity Score	Docking Score		Glide Energy		Glide Emodel	
			Glide XP	IFD	Glide XP	IFD	Glide XP	IFD
1	ZINC49069570	0.815	−6.124	−13.022	−46.239	−65.392	−60.563	−88.454
2	ZINC49115270	0.813	−5.901	−12.832	−42.167	−66.115	−57.363	−90.390
3	ZINC14436185	0.752	−7.634	−12.797	−42.616	−64.565	−57.146	−91.931
4	ZINC14642643	0.714	−6.561	−12.768	−44.956	−67.071	−59.660	−81.700
5	NCI-714028	0.782	−7.065	−12.570	−41.890	−47.369	−58.586	−78.464
6	ZINC03978503	0.730	−6.786	−12.235	−43.772	−70.751	−52.900	−98.167
7	ZINC06040160	0.732	−7.151	−12.075	−40.903	−61.458	−56.222	−92.196
8	ZINC84428482	0.774	−5.161	−11.729	−41.450	−69.406	−46.531	−103.352
9	ZINC03870412	0.783	−6.776	−11.639	−38.114	−60.696	−54.416	−77.114
10	NCI-636594	0.737	−5.806	−11.597	−41.641	−57.405	−58.223	−86.238
11	ZINC85648240	0.754	−5.379	−11.436	−43.130	−68.177	−57.735	−93.966
12	EGCG	—supplementary information whereas	−5.771	−11. 352	−45.549	−72.514	−56.194	−79.541

### Molecular interactions with the screened compounds in comparison with the reported EGCG

Further, the prepared three dimensional structure of the oncoproteins namely E5, E6 & E7 and other proteins namely Aromatase and ERα obtained from the homology modeling as well as the crystal structure has been taken forward for the molecular docking studies with shape screened compounds and EGCG which is a query structure. The validation of the modeled E5 structure will be represented in the Supplementary information. The conformation and orientation of the binding site within the target were envisaged with the help of docking process. The extra precision (XP) docking procedure was favored since this program considers the non-favourable interactions and penalizes them which turn out to be more precise than standard precision (SP) docking. The binding site of E5 & E6 oncoproteins were predicted with the help of Schrödinger suite whereas for E7 as we have mentioned in our earlier work^8^. The docking site for aromatase and ERα were predicted with the available co-crystallized ligand and used for further docking strategies.

It was clearly evident from the reports that EGCG possess better inhibitory activity towards E5, E6 & E7 which is the oncoprotein of HPV causing cervical cancer. Qiao *et al*., stated that EGCG also suppress the expression of aromatase and ERα in cell lines of cervical cancer which is indirectly responsible for the higher expression of E6/E7 which is vital in cell proliferation. Hence, we investigated the inhibitory activity of EGCG and its analogues for E5, E6, E7, aromatase & ERα through docking studies. A total of 11 ligand molecules obtained from the shape screening protocol and the reference ligand, EGCG were docked on to the active site of HPV 16 E5, E6, E7, aromatase & ERα. The docking results indicated that EGCG and its analogues exhibited good docking scores and with all the oncoproteins, aromatase & ERα during extra precision docking whereas the interactions were slightly in dejection except E7. Since, E7 plays a significant role in transforming activity including disruption of normal epithelial differentiation and proliferation allowing viral replication and carcinogenic transformation^14^ we have concentrated on E7 activities and its inhibition.

Followed by glide docking, Induced fit docking protocol is also included in the study for E7 oncoprotein which is helpful in the identification of binding mode in various flexible conformations. Consequently, in the current study, the chosen 11 ligands were subjected to the extra precision docking and induced fit docking protocol for E7 oncoprotein. For the E7 oncoprotein the XP docking scores were ranging from −7.634 kcal/mol to −5.161 kcal/mol whereas the IFD docking scores ranged from −13.022 kcal/mol to −11.352 kcal/mol. The docking results of E5, E6, E7, aromatase &ERα with EGCG and its analogues were represented in Supplementary Table 2. With respect to the docking scores of E7 oncoprotein, it was observed that the IFD protocol has shown higher scores than XP for the molecules which is evident from Table 2. It also states that apart from docking scores, glide energy and glide e-model values, the interactions between receptor - ligand are also better in IFD than XP. Among all the ligand molecules, we observed a total of five compounds from the ZINC database and NCI library to be more potent based on the docking score and the visual inspection of the interaction.

**Table 2:** A summary of interacting amino acid residues with the best identified small molecule ligands under study upon Glide XP docking and Induced fit docking.

S.No	Compound Name	Glide XP Docking		Induced Fit docking	
		Interacting Residues	Distance of the interaction	Interacting Residues	Distance of the interaction
1	ZINC49069570	Asp 75	Asp 75 – A (1.83)	Thr 72	Thr 72–A (1.77)
		Val 74	Val 74 – D (2.65)		Thr 72–A (1.79)
		Glu 18	Glu 18 – A (1.94)	Thr 19	Thr 19–A (1.78)
		Thr 19	Thr 19 – A (1.70)	Ser 71	Ser 71–D (1.77)
				Glu 18	Glu 18–A (1.90)
					Glu 18–A (2.20)
					Glu 18–D (1.99)
2	ZINC49115270	Val 74	Val 74 – A (2.13)	Thr 19	Thr 19–A (1.85)
		Thr 19	Thr 19 – A (1.84)	Gln 96	Gln 96–D (2.25)
		Asp 75	Asp 75 – A (1.64)	Glu 18	Glu 18–A (2.05)
			Asp 75 – A (2.16)		Glu 18–A (2.62)
				Thr 72	Thr 72–A (1.65)
					Thr 72–A (1.92)
				Ser 71	Ser 71–D (1.82)
					Ser 71–D (2.63)
3	ZINC14436185	Thr 20	Thr 20 – A (1.91)	Thr 78	Thr 78–A (1.88)
		Thr 72	Thr 72 – A (2.18)	Leu 79	Leu 79–A (2.63)
		Glu 18	Glu 18 – A (1.97)	Glu 18	Glu 18–D (2.24)
			Glu 18 – A (1.88)	Ser 71	Ser 71–D (1.84)
				Thr 20	Thr 20–D (1.95)
				Thr 72	Thr 72–A (2.02)
					Thr 72–A (1.76)
4	ZINC14642643	Thr 20	Thr 20 – A (1.89)	Thr 72	Thr 72–A (1.92)
		Thr 72	Thr 72 – A (2.03)	Asp 75	Asp 75–A (2.07)
		Thr 78	Thr 78 – A (1.52)	Glu 18	Glu 18–A (1.53)
		Glu 18	Glu 18 – A (2.18)		Glu 18–A (1.90)
			Glu 18 – A (1.99)	Thr 20	Thr 20–A (2.52)
			Glu 18 – A (1.80)		Thr 20–D (1.69)
5	NCI-714028	Thr 78	Thr 78 – A (1.77)	Glu 18	Glu18–A (1.93)
			Thr 78 – A (2.03)		Glu 18–A (1.81)
		Thr 20	Thr 20 – A (1.92)		Glu 18–D (2.09)
		Thr 72	Thr 72 – A (2.09)	Val 74	Val 74–D (2.04)
		Glu 18	Glu 18 – A (1.68)		Val 74–D (2.62)
				Asp 75	Asp 75–A (1.76)
				Thr 19	Thr 19–A (1.93)
				Gln 96	Gln 96–A (2.09)
6	EGCG	Glu 18	Glu 18 – A (1.97)	Val 74	Val 74–D (2.41)
		Gln 96	Gln 96 – A (2.10)	Asp 75	Asp 75–A (1.60)
		Val 90	Val 90 – A (2.14)	Thr 19	Thr 19–D (2.75)
			Val 90 – A (2.49)	Glu 18	Glu 18–A (1.73)
				Thr 72	Thr 72–A (2.23), Thr 72–A (1.84)

*A- Acceptor *D-Donor.

**Figure 2:**
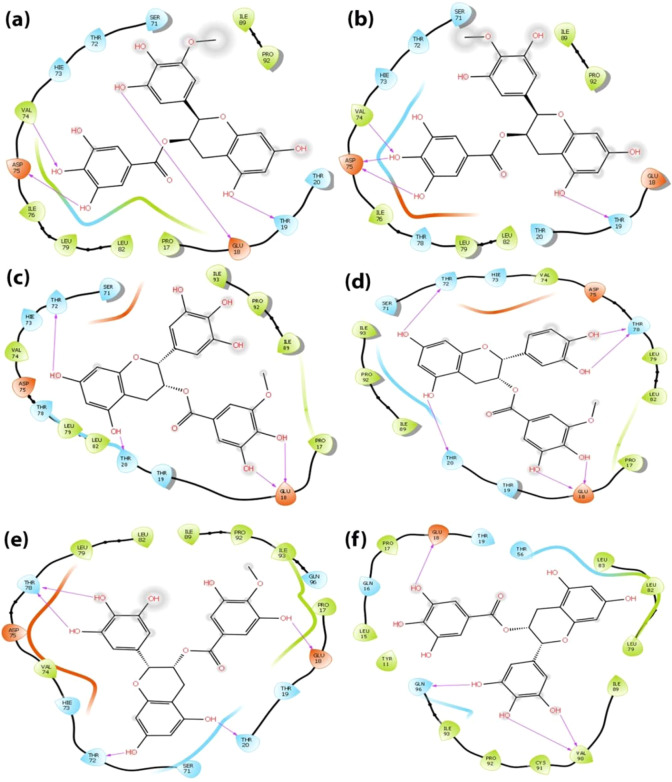
2 Dimensional Interaction diagram of the best identified small molecule compounds obtained during the Glide XP docking.

**Figure 3:**
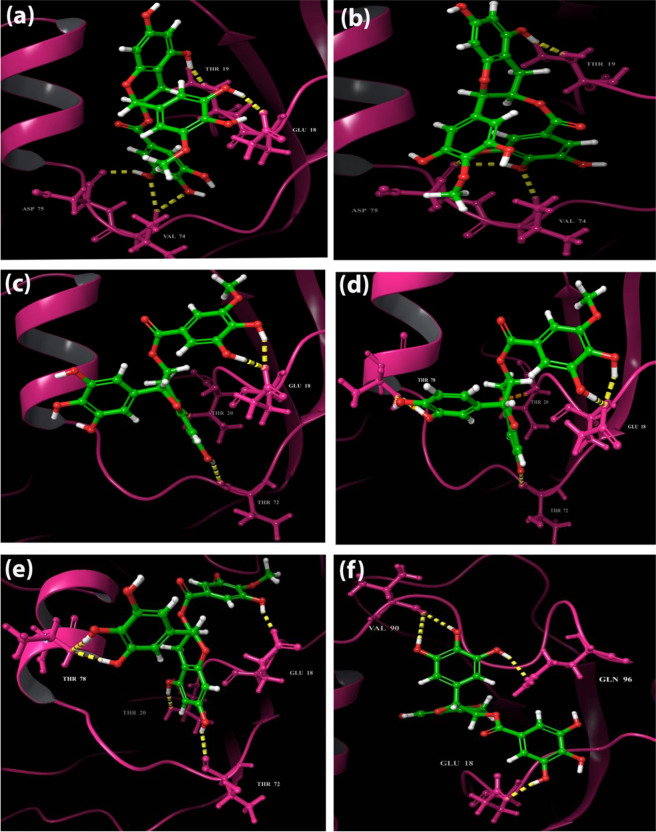
Binding pocket amino acid residue interaction patterns of bound best identified small molecule compounds during the Glide XP docking. Hydrogen bonds are depicted by a yellow dotted line.

**Figure 4:**
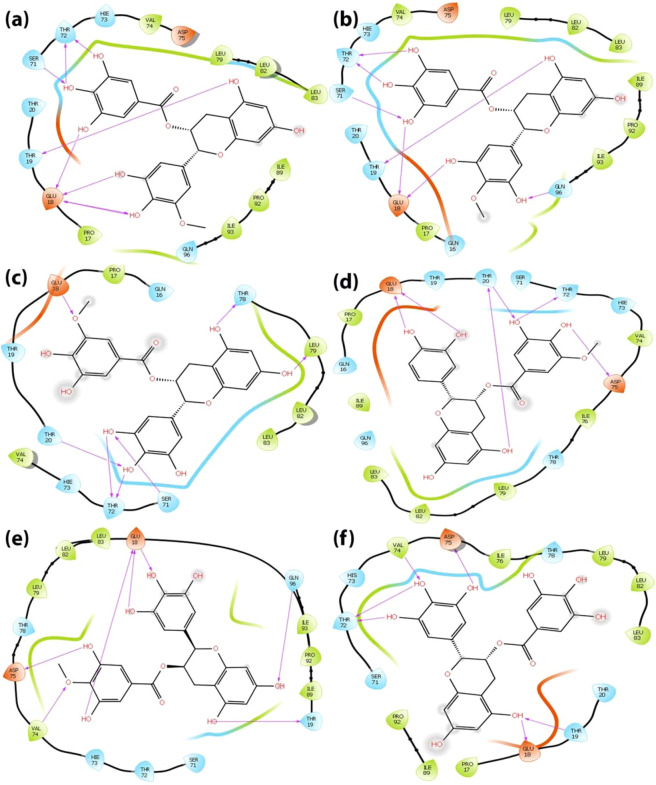
2 Dimensional Interaction diagram of the best identified small molecule compounds obtained during the Induced Fit docking.

**Figure 5:**
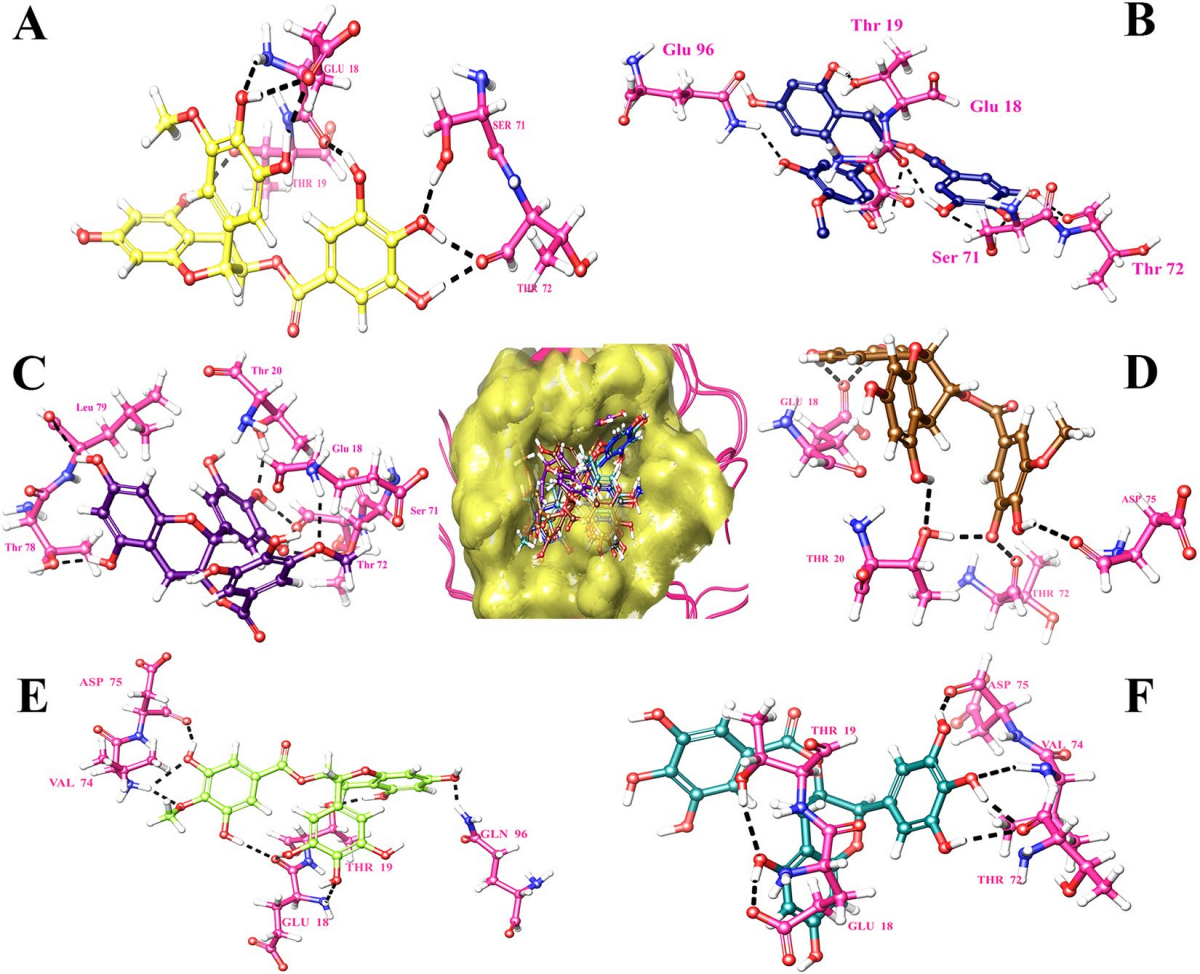
Binding pocket amino acid residue interactions patterns of bound best identified small molecule compounds during the Induced Fit docking. Hydrogen bonds are depicted by a yellow dotted line.

Analysis of the binding mode of the identified compounds through Glide XP docking revealed that the interactions are among the structured region CR3 of HPV 16 E7 which is evident through 2D interaction diagram and the docking pose of the identified small molecuels represented in Figs. 2 and 3 respectively. The 2D interaction diagram and the docking pose of the induced fit docking practice is represented in Figs. 4 and 5 and it clearly shows that induced fit docking approach helps in obtaining the better interaction and energy profiles. Table 2 illustrates the interaction profiles and distance between the compound and receptor obtained through both the docking strategies of top five compounds and EGCG. The best five identified small molecule compounds from ZINC database (ZINC49069570, ZINC49115270, and ZINC14436185 and ZINC14642643), NCI library (714028) and EGCG were identified to be prominent inhibitor of the HPV 16 E7 oncoprotein. The residues of the HPV 16 E7 binding pocket such as Glu 18, Thr 19, Thr 20, Thr 72, Val 74, Asp 75, Thr 78 and Gln 96 were identified to show stable hydrogen bonding with the above mentioned compounds. Figs. 2 and 4 represents the 2D interaction diagram of the top identified small molecules carried forward for the Glide XP and IFD protocol along with EGCG in order to compare the binding mechanism between the available and identified ligands. Fig. 2 represents the Glide XP study and it shows that the mostly hydrogen bond interactions are present and the residue Glu 18 is found interacting with all the identified small molecules whereas Thr 72, Val 74, Asp 75 and Thr 78 found interacting to most of the identified small molecules. It is clearly evident from the Fig. 4 that the induced fit docking also states that hydrogen bond interactions were mostly observed and the residues Glu 18 and Thr 72 were observed to interact with all the compounds and notably the residue Thr 19, Thr 20, Val 74 and Asp 75 interacts with most of the identified small molecules. Figs. 3 and 5 represents the 3D interaction of the top identified small molecules of Glide XP and IFD respectively. The binding mode analyses of the top two compounds among the six compounds and EGCG with the oncoprotein were described in detailed. The interaction profiles of the E5, E6, aromatase and ERα were represented in Supplementary Figures No 3, 4, 5 and 6.

#### Binding mode of the compound ZINC49069570 with the E7 oncoprotein

The compound ZINC49069570 has observed to possess docking score of −6.124 kcal/mol, glide energy of −46.239 kcal/mol and glide emodel of −60.563 kcal/mol during the extra precision docking and the hydrogen bond interactions between the compound were observed with the residues Asp 75, Val 74, Glu 18 and Thr 19. The interactions during extra precision were represented in the Figs. 2(a) and 3(a). During Glide extra precision docking the protein is rigid whereas in induced fit docking protocol, the protein and ligand both becomes flexible and hence the protein shows difference in the interaction with amino acids. When the induced fit docking strategy has been applied an increase in the docking score to −13.022 kcal/mol, glide energy to −65.392 kcal/mol and the glide emodel to −88.454 kcal/mol is obtained stating the flexible conformations and the more number of interactions. The hydrogen bond interactions during induced docking were identified with the backbone amino acid residues Thr 72, Thr 19, Glu 18 and Ser 71. All the hydroxyl group of the benzene ring forms h-bond interaction with the atom OH and O of the backbone residues of Ser 71, Thr 72 and Glu 18 with the distance of 1.79 Å, 1.77 Å and 1.90 Å respectively whereas the hydroxyl group of the methoxybenzene ring forms h-bond interaction with the amino group and oxygen of the backbone residue Glu 18. Further, the hydroxyl group of the benzopyran ring forms h-bond interaction with the OH group of the residue Thr 19 with the distance of 1.78 Å. The 2D interaction diagram of the docked complex obtained during IFD is represented in the Fig. 4(a) and the 3D docking pose of the docked complex is represented in the Fig. 5(A) represented in colour yellow for compound.

#### Binding mode of the compound ZINC49115270 with the E7 oncoprotein

The compound ZINC49115270 binds well with the E7 oncoprotein and represented a docking score of −5.901 kcal/mol, glide energy of −42.167 kcal/mol and emodel of −57.363 kcal/mol during the extra precision docking. The interactions observed with the E7 oncoprotein states that, it is devoted to be residues Val 74, Thr 19 and Asp 75. The residue which interacts during extra precision is also present during IFD along with other interacting residues stating that IFD posses additional interactions. The compound ZINC49115270 was identified with the docking score of −12.832 kcal/mol, glide energy of −66.115 kcal/mol and the glide emodel of −90.390 kcal/mol during the induced fit docking strategy. The hydrogen bond interactions were formed between the compound and the backbone residues Ser 71, Thr 72, Glu 18, Thr 19 and Gln 96. The interactions were focused between the hydroxyl group of the benzene ring in the compound and the oxygen of the Thr 72 with the distance of 1.65 Å and 1.92 Å respectively whereas the hydroxyl group of the benzene ring forms h-bond interaction with the OH group of the Ser 71 with the distance of 1.82 Å and 2.63 Å respectively. Further, the amino group of the backbone residue Gln 96 forms h-bond interaction with the hydroxyl group of the methoxybenzene ring with the distance of 2.25 Å. Additionally, the hydroxyl group of the benzopyran ring forms the h-bond interaction with the backbone residue Thr 19 with the distance of 1.85 Å. These interactions were evident with the 2D interaction diagram represented in Fig. 4(b) and the 3D docking pose of the docked complex is represented in Fig. 5(B) represented in colour navy blue for compound.

#### Binding mode of the compound EGCG with the E7 oncoprotein

The compound EGCG has shown the energy of extra-precision docking to be −5.771 kcal/mol and glide emodel score of −56.194 kcal/mol during the extra precision docking and the hydrogen bond interactions developed between the compound and the protein were observed to be Glu18, Gln 96 and Val 90 evident with the Figs. 2(f) and 3(f). Whereas, the compound EGCG which is already reported for the cervical cancer cell lines has acquired an increase in the docking score to −11.352 kcal/mol during the induced fit docking strategy with the glide energy of −72.514 kcal/mol and glide emodel of −79.541 kcal/mol. Further, the hydrogen bond interactions were formed between the compound and the backbone residues Val 74, Asp 75, Thr 19, Glu 18 and Thr 72. The hydroxyl group of benzene ring interacts with the oxygen of the backbone residue Thr 72 and Asp 75 with the distance of 1.89 Å and 1.60 Å respectively whereas the amino group of Val 74 interacted with the hydroxyl group of benzene ring with the distance of 2.41 Å. On the other hand, the hydroxyl group of benzopyran ring in the compound interacts with the carboxyl group of the backbone residue Glu 18 with the distance of 1.73 Å and the hydroxyl group of the backbone residue Thr 19 with the distance of 2.75 Å. These interactions were evident with the 2D interaction diagram and the docking pose of the compound which is represented in the Figs. 4(f) and 5(F) respectively.

### ADME properties prediction

The identified small molecules and the reference molecule EGCG were further subjected to the evaluation of physicochemical and biological features since it is very essential due to the implementation of the combinatorial chemistry and high throughput screening. Molecular weight, volume, QPlogPo/W, QPlogPw, QPlogHERG and percentage human oral absorption were the parameters chosen for the analysis and the predicted values are represented in Table 3. The molecular weight ranges between 440 and 500 whereas the volume is defined as the total solvent-accessible volume and it ranges from 1200 to 1300 Å^3^. All the predicted values of the descriptors falls within the accepted range for drug-likeness, thus bearing positive indications that the identified lead molecules are promising drug like entities.

**Table 3:** ADME analysis of the Top 5 screened compound and the reference molecule EGCG.

S.No	Compound Name	MW^a^	Volume^b^	Donor HB^c^	Acceptor HB^d^	% Human Oral absorption^e^	QPlogPo/w^f^	QPlogHERG^g^
1	ZINC14436185	472.404	1282.640	6	8	39.172	0.629	−5.563
2	ZINC49069570	472.402	1294.223	6	11	39.172	0.524	−5.478
3	ZINC49115270	471.394	1284.549	6	11	37.582	0.590	−5.612
4	ZINC14642643	456.405	1261.942	6	8	34.156	1.207	−5.520
5	NCI-714028	442.378	1224.927	7	8	24	0.428	−5.715
6	EGCG	458.375	1255.754	8	8	1.378	−0.262	−5.610

^a^Molecular Weight of the molecule (accepted range: 130.0–725.0).

^b^Total sovent-accessible volume in cubic angstroms using a probe with a 1.4 Å radius (acceptable range: 500.0–2000.0).

^c^Estimated number of hydrogen bonds that would be donated by the solute to water molecules in an aqueous solution (range: 0.0–6.0).

^d^Estimated number of hydrogen bonds that would be accepted by the solute from water molecules in an aqueous solution (accepted range: 2.0–20.0).

^e^Percentage Human Oral Absorption(acceptable range: <25% is poor and >80% is high).

^f^Predicted octanol/water partition co-efficient log p (acceptable range: −2.0 to 6.5).

^g^Predicted IC50 value for blockage of HERG K+ channels (concern below −5.0).

### Binding free energy calculation

In the current study, the evaluation of molecular docking with the related post-scoring approach, MM-GBSA is reported for the complexes with HPV 16 E7. The results from the free energy of binding prediction using MM-GBSA is listed in Table 4 and it helps to gain further insights into the interaction between HPV 16 E7 and the ligands. This calculation of binding energy states that the docked pose shows better binding energy and the binding affinity. The top five compounds obtained from the shape screening and the reported EGCG were utilized for the binding free energy analysis and possess binding energy to the range of −92.427 kcal/mol to −81.522 kcal/mol which is evident in the Table 4. It is also observed that the binding free energy of the complexes were well correlated with the docking scores and according to the energy components of the binding free energies, the major favourable contributors to ligand binding are the van der Waals and Coulomb energy which is the major driving force. It is clearly apparent with the binding free energy calculation that the compounds obtained from the shape screening protocol similar to EGCG portrays that they bound more strongly to E7 oncoprotein than the reference ligand (EGCG). There arises a conclusion that there is a possibility that these compounds can be a potent inhibitor to inhibit the E7 protein than EGCG.

**Table 4:** Binding free energy calculation of the best identified small molecule compounds that interacts with HPV E7 oncoprotein.

S.No	Compound Name	ΔG_bind_	ΔG_covalent_	ΔG_couloumb_	ΔG_H-bond_	ΔG_solvGB_	ΔG_vdW_
1	ZINC49069570	−92.427	−52.967	2.499	−3.310	23.607	−30.445
2	ZINC49115270	−91.104	−33.380	7.018	−1.727	20.266	−43.985
3	ZINC14436185	−85.354	−46.089	1.531	−1.781	21.232	−29.216
4	ZINC14642643	−82.581	−45.708	5.217	−2.983	25.145	−34.372
5	NCI-714028	−81.522	−48.767	2.201	−2.294	27.057	−29.242
6	EGCG	−84.723	−31.857	2.974	−1.468	26.428	−43.425

### Electronic structure calculations for the observed best compounds

**Figure 6:**
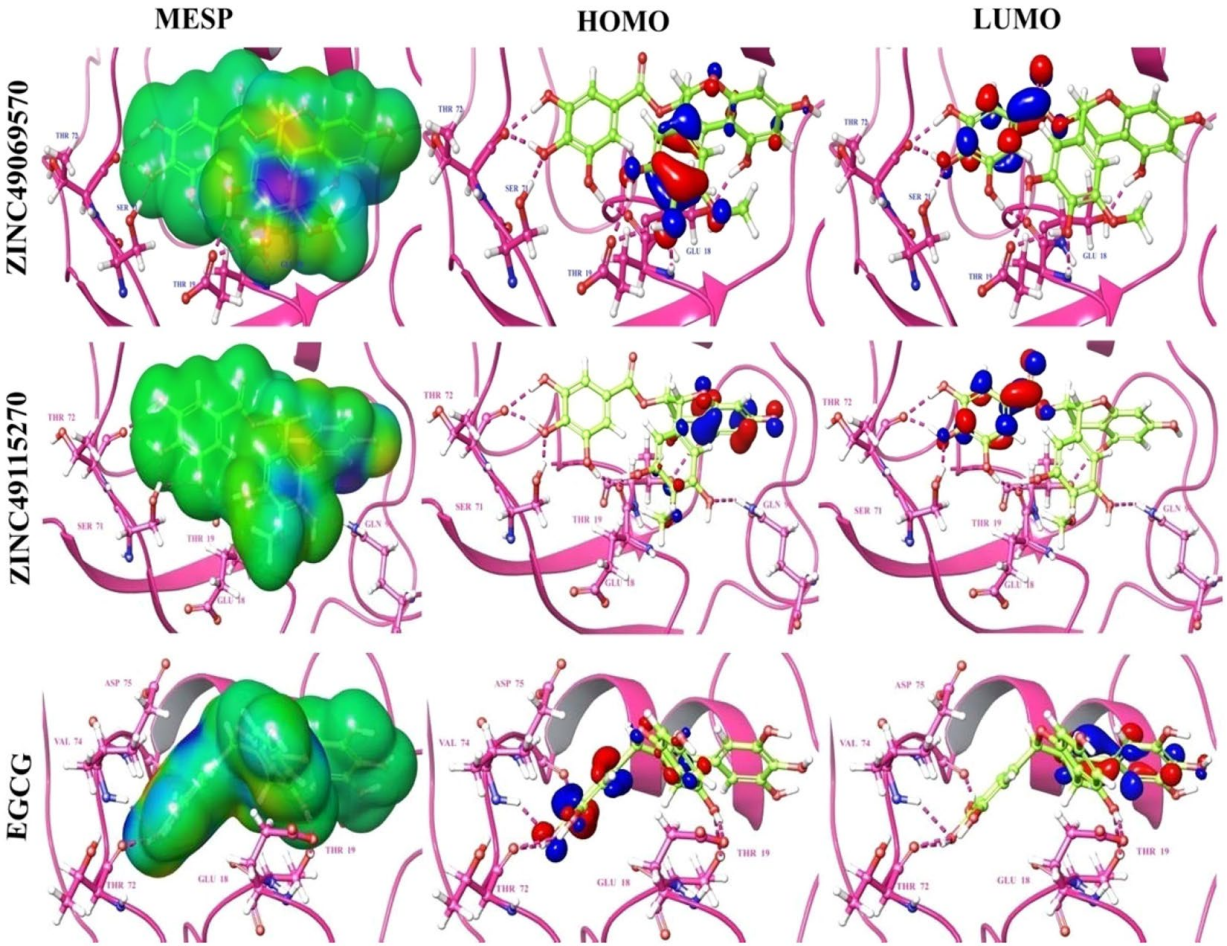
Electronic structure calculation studies of the best identified small molecule compounds and the reference compound EGCG.

The identified molecules and the reference compound EGCG were optimized using the hybrid DFT inclusive of the Becke’s three parameter exchange potential and the Lee yang-Parr Correlation functional theory with the 6–31 G** basis set in Jaguar. The MESP calculation, HOMO, LUMO energy calculation and the band gap analysis were carried out to represent the electron transfer which contributes enormously in binding of the compound with the target. The molecular electrostatic surface potential associates the total charge distribution with the dipole moment, electron negativity, partial charges and the site of chemical reactivity of the molecules. The electrostatic potential at the surface represented in Fig. 6 for the compounds ZINC49069570, ZINC49115270 and EGCG possess attractive potential represented in red coloured regions whereas the repulsive potential appears to be in blue and the region of moderate potential is represented in green. The repulsive potential corresponds to the repulsion of electron and the nuclear charge is completely shielded. It is clearly evident with the MESP representation of the compounds in Fig. 6 that the positively charged region of the compounds plays an important role in binding to the target. Also, the electrostatic potential states that there acquires the electropositive potential region over the oxygen atom and the most neutral region over the other part of the compounds. Thus, the electrostatic potential of the inhibitors plays a significant role in the interaction and consequently influence the inhibition effect. Table 5 summarizes the theoretical electrostatic parameters of the compounds. The values of HOMO and LUMO were ranging between −0.215583 to −0.053442 eV. The HOMO energy demonstrate the ionization potential of the identified small molecules where as the LUMO energies depict the electron affinity of the identified small molecules which possess a positive value that illustrates the capacity to accept electrons and forms strong interaction with the protein. The small energy gap of HOMO and LUMO for screened compounds varies between 0.266033 and 0.270687. This strongly states that there is less stability and makes electron transfer to be rapid and exchange equally to make these compounds reactive. The screened compounds possess higher HOMO-LUMO band gaps than EGCG which proposes them to be more polarizable. Hence, the analysis observed states that the screened molecules retrieve the ability to form stronger interactions without any covalent modification of the proteins. The HOMO-LUMO gap signifies the chemical stability of the molecules since they relate the specific movement of electrons. The compound ZINC49069570 possess higher HLG value stating that the compound is reactive when compared with the other compounds which directly states the impact on the inhibitory activity against the HPV oncoprotein.

**Table 5:** Electronic structure analysis of the best identified small molecule compounds and the value of the forniter orbital.

S.No	Compound Name	HOMO (eV)	LUMO (ev)	HLG (eV)
1	ZINC49115270	−0.217867	−0.052820	0.270687
2	ZINC49069570	−0.215583	−0.053442	0.269025
3	EGCG	−0.215979	−0.050054	0.266033

### Binding conformation stability analysis

**Figure 7:**
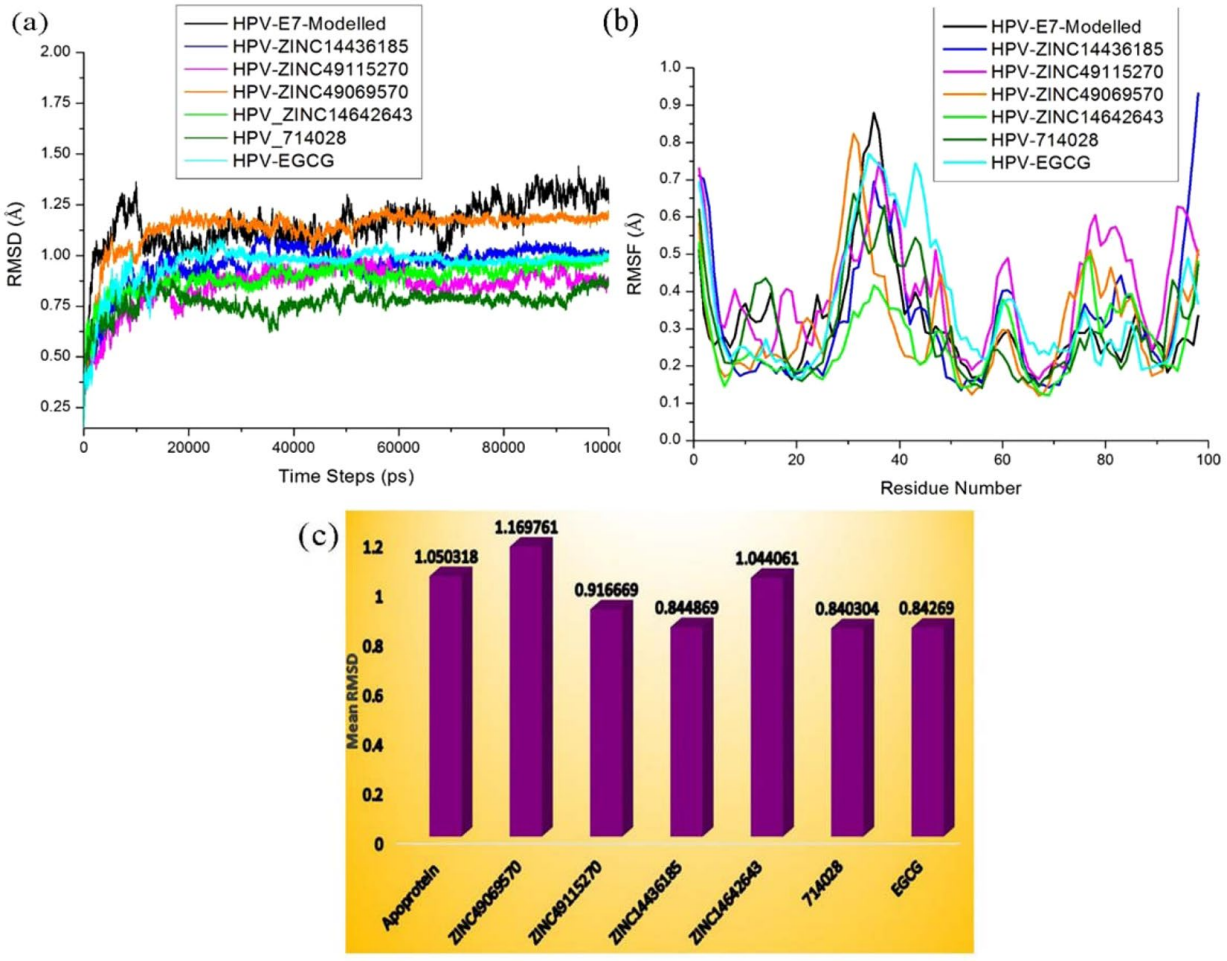
(**a**) RMSD plots of the best identified small molecule compounds and EGCG with the HPV E7 oncoprotein (**b**) RMSF plots of the best identified small molecule compounds and EGCG with the HPV E7 oncoprotein (**c**) Mean RMSD of the best identified small molecule compounds and EGCG with the HPV E7 oncoprotein.

**Figure 8:**
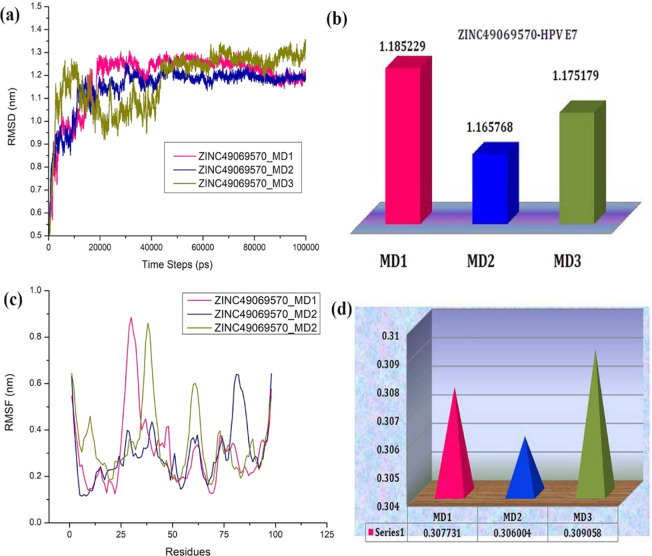
(**a**) RMSD plots of the best identified small molecule compound ZINC49069570 with the HPV E7 oncoprotein for three sets of simulation (**b**) Average Mean of the RMSD plots of the best Identified ZINC49069570 with the HPV E7 oncoprotein for three sets of simulation. (**c**) RMSF plots of the best identified small molecule compound ZINC49069570 with the HPV E7 oncoprotein for three sets of simulation (**b**) Average Mean of the RMSF plots of the best Identified ZINC49069570 with the HPV E7 oncoprotein for three sets of simulation.

**Figure 9:**
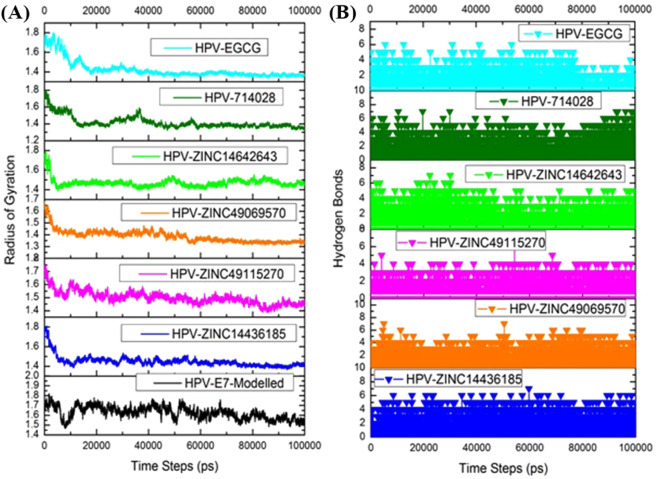
(**A**) Radius of Gyration plots of the best identified small molecule compounds and EGCG with the HPV E7 oncoprotein (**B**) Hydrogen Bond analysis of the best identified small molecule compounds and EGCG with the HPV E7 oncoprotein.

The binding stability of the ligand in the active site of the target and the behaviour of the protein in dynamic environment can be studied using the molecular dynamics simulation. In this study, the molecular dynamics simulation has been carried out for 100 ns simulation using GROMACS. The dynamics simulation has been performed twice and on the whole three sets of simulation has been carried out which helps in depicting the statistical significance of the results obtained. The best docking poses of the protein with the compounds ZINC49069570, ZINC49115270, ZINC14436185, and ZINC14642643 from ZINC database, 714028 from NCI library and EGCG from the literature has been used as input for molecular dynamics simulation along with the modelled HPV 16 E7 oncoprotein in the current study. We have endeavored to compare the MDS behaviour of the complexes generated using the screened and reference compound. The changes in the structural features have been analyzed through the calculation of the root mean square deviation (RMSD) and the root mean square fluctuation (RMSF) over the back bone atoms. The Root Mean square deviation (RMSD) obtained during simulation 1 is depicted in the Fig. 7(a) which shows that there is no much deviation throughout the simulation time for the ligand molecules except the modelled structure. The graphs indicated that all the compounds were better equilibrated and stabilized than the modelled structure after a period of 70 ns. However, the modelled structure have slight deviations but attained its stability around 1.30 Å after 85 ns. The complexes have initial deviations which were considered to be the time taken for equilibration and the rmsd value describes that the complexes attained its stability at 1.10 Å. The six compounds have deviations till 40 ns simulation but later the complexes attained its stability throughout the simulation. The compound ZINC49069570 which is observed to be the best identified small molecule and highly similar to the reference compound evident with similarity score of 0.876 is represented in Fig. 7(a) with colour orange showing considerable deviations till 40 ns time period. The compound further maintained its stability after 80 ns at 1.10 Å whereas the Root Mean Square Fluctuation (RMSF) of the compound depicted in Fig. 7(b) shows that the fluctuation is observed in the loop region with the residues 25–40. Slight fluctuations that is observed thereafter does not affect the binding of the ligand and the conformational stability. The RMSD graph has been supported by the RMSF graph which represents the fluctuation of all the residues. The compound ZINC49115270 is observed to be the next best identified small molecule and it is represented in colour magenta in Fig. 7(a). This shows that the compound deviated upto 1.0 Å till 40 ns and maintained its stability after 58 ns till the completion of simulation at 100 ns. When the RMSF analyses have been observed for the same compound in Fig. 7(b), it states that there occurs minimal fluctuation at the loop region which is denoted be disordered^22^. The other compounds 714028, ZINC14436185, ZINC14642643 and EGCG also shows that there are slight deviations when compared to the best identified two small molecules. When the screened compounds are analyzed in comparison with EGCG, it denotes that the screened ZINC49069570 and ZINC49115270 possess better stability and does not have any problem with the binding of the protein. When the mean RMSD is observed for the top compounds and apoprotein, it is clearly evident with the Fig. 7(c) that the mean value corresponds to the deviations of the protein. However, these fluctuations were observed in the loop region and the binding of protein with the ligand molecule is not much affected during the simulation period. It has been observed clearly from the results that the analysis of dynamic simulation performed once portrays it to be difficult to access the stability and conformational behavior and it does not helps in providing the statistical significance. Hence, in order to clearly understand the dynamic behavior of the protein and provide the statistically valid belief on the molecular dynamics simulation, the simulation has been repeated for three times with the same parameters. The results has been interpreted for the simulation performed thrice with the average mean for the triplet dynamics along with the root mean square deviation and fluctuation of the complex HPV E7 – ZINC49069570 in Fig. 8 stating that the difference were observed to be in 0.01 Å between each other. The simulation results of the triplicate dynamics has been represented in Supplementary information Fig. 7. The difference observed in all the three types of simulation for the complex HPV E7 – ZINC49069570 has been provided in Fig. 8(a) as RMSD stating that the slight deviations are available between each dynamics study and Fig. 8(b) depicts the average mean obtained from RMSD of the three simulation representing the 0.01Å difference between each simulation. Fig. 8(c,d) represents the root mean square fluctuation observed for the complex of the protein with ZINC49069570 and average mean for the RMSF respectively. The radius of gyration graph represented in the Fig. 9(A) shows the compactness of the protein and this supports that the RMSD graph having slight deviations in all complexes throughout the simulation period except the screened compound ZINC49069570, ZINC49115270 and the reference EGCG molecule. In Fig. 9(B) we observed hydrogen bond interaction between the protein and the ligand molecules of simulation 1 which states that during the simulation period the compound ZINC49069570 and ZINC49115270 does not loses any interaction with the protein which is formed during the docking strategy and the interaction has been maintained during the course period of simulation. But when we analyses the other compounds, it is evident that there is some loss of interaction but regained during the simulation period which is not very strong enough. The RMSF, RMSD and H-bond for all the complexes obtained during the repeats of simualtion has been incorporated in Supplementary Figures 7, 8 and 9 respectively. From the results in Supplementary Figure 7, it is clearly evident that no differences were observed for the simulation performed. It also states that the results has been valid statistically and no significant changes has been obtained. Also, the simulation has been performed with the same system setup which helps to conclude that the results are obtained with the robust analysis on the statistical significance.

These analyses suggested that the compounds ZINC49069570, ZINC49115270 identified which is analogue to EGCG can be potent inhibitors in comparison to ZINC14436185, ZINC14642643 and 714028 against E7 CR3 region and could play a noteworthy role in the development of drugs.

### Post dynamics binding free energy calculation

**Figure 10:**
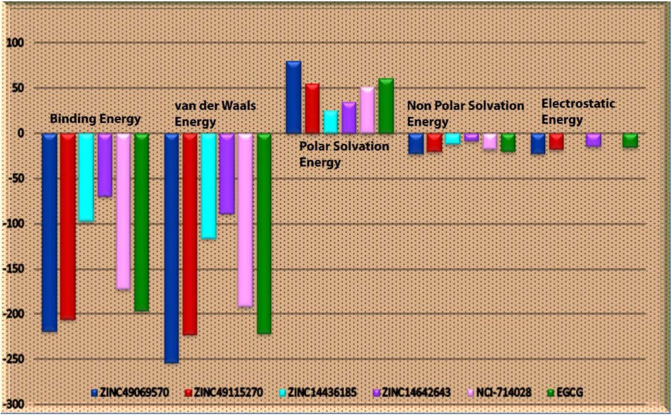
Graph showing the distribution of the various interaction energies like binding free energy, van der Waals energy, polar solvation, non-polar solvation energy and Electrostatic energy of the identified ligands and reference molecule.

The most recently used methods for analysis of the binding free energy calculation after the molecular dynamics simulation with the trajectory obtained is named as MM/PBSA approach. This method will be very helpful in the calculation of interaction energies and is frequently engaged to study biomolecular complexes. The g_mmpbsa has been demonstrated to be an operational tool employed to estimate the free energy of relative binding as well as provide a breakdown of residue influence to binding. Around 200 snapshots were mined at every 500 ps of constant intervals throughout the simulation trajectory. The free energy of binding and its consistent components acquired from the MM/PBSA calculation of the HPV 16 E7- drug like inhibitor complexes are enumerated in Table 6. It is clearly evident with the results that, the compounds ZINC49069570 and ZINC49115270 have higher binding energies when compared with the other inhibitor and the reference EGCG molecule. The values obtained indicate the contribution of interaction dominated by van der Waals among the interaction energy was much larger than the other energies. The distribution of various interaction energies obtained through binding free energy calculation were represented in Fig. 10. This states that the energies of the compound ZINC49069570 and ZINC49115270 throughout the molecular dynamics simulation is high indicating the strength of binding. The value of the solvation energy specified little involvement to the ligand binding with HPV 16 E7 is very strong and it was found according to free energy calculations, hydrophobic contacts are a key to HPV E7 inhibition by this series of inhibitors.

**Table 6:** MM/PBSA free energy of the compounds that interacts with HPV E7 oncoprotein calculated from the MD simulations.

S.No	Compound Name	Binding Energy	van der Waal Energy	Polar Solvation Energy	Non-polar solvation energy	Electrostatic Energy
1	ZINC49069570	−217.957 ± 38.974	−253.404 ± 43.658	80.221 ± 20.942	−22.147 ± 3.625	−22.626 ± 11.676
2	ZINC49115270	−205.563 ± 58.151	−222.687 ± 58.069	55.121 ± 20.946	−20.453 ± 5.127	−17.544 ± 15.166
3	ZINC14436185	−97.011 ± 85.684	−115.286 ± 99.415	25.380 ± 29.866	−11.392 ± 9.924	3.287 11.349
4	ZINC14642643	−69.444 ± 105.707	−88.395 ± 119.783	34.281 ± 42.254	−7.869 ± 10.545	−14.381 ± 16.221
5	NCI-714028	−171.948 ± 88.498	−191.038 ± 94.425	51.503 ± 30.856	−18.032 ± 8.928	−7.640 ± 12.230
6	EGCG	−195.265 ± 70.833	−220.817 ± 74.303	61.720 ± 24.781	−20.441 ± 6.856	−15.788 ± 14.959

### Principle Component Analysis

**Figure 11:**
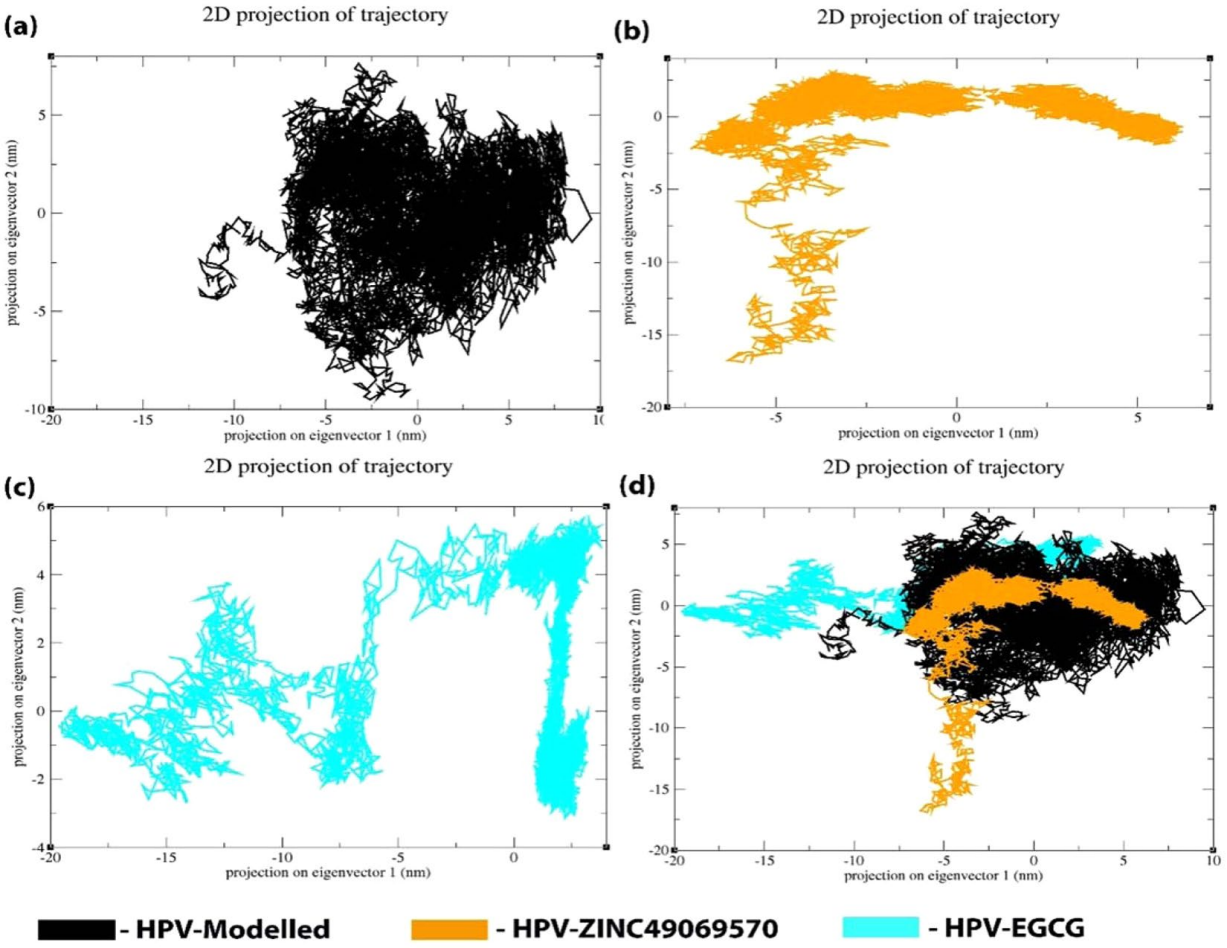
Principle component analysis of the Modelled HPV E7 oncoprotein, HPV ZINC49069570 complex and HPV EGCG complex represented in Black, Orange and Cyan respectively.

Eigen vectors with the largest associated eigen values define the essential subspace in which most of the protein dynamics occurs. The cluster of stable states can be visualized with the help of these projections and two features are very apparent from these plots. Firstly, the clusters are better defined in the identified small molecule than the reference molecule EGCG. Secondly, the reference compound EGCG covers a wide region of phase space especially along the PC2 plane when compared to the compound ZINC49069570 which is evident with the Fig. 11(c). In Fig. 11 it is clear that the projection of the two eigenvectors of HPV E7 complex with the lead ZINC49069570 and EGCG reveals that the apoprotein and HPV-EGCG explores a large conformational space in comparison to the HPV-ZINC49069570 which displays the minimum conformational space. When the complex HPV-EGCG is observed, it portrays the slight increase in the conformational space when compared with the HPV-ZINC49069570. The overall flexibility of the protein complexes were examined by the trace of diagonalized covariance matrix of the Cα atomic positional fluctuations. A significant compactness was observed in the HPV-ZINC49069570 complex facilitating vital interactions at 300 K.

### Secondary structure analysis during the simulation studies

**Figure 12:**
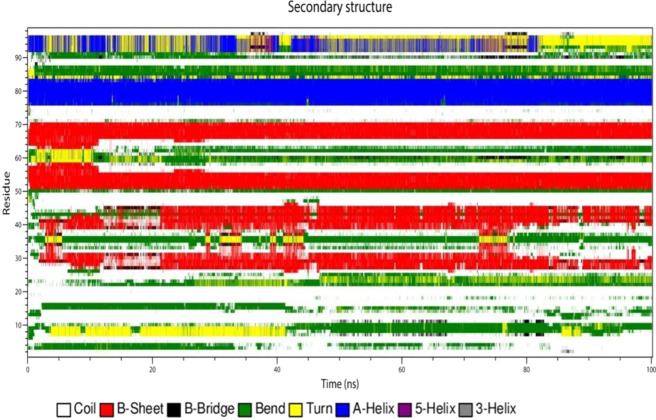
Conformational evolution of secondary structure elements of HPV E7 modelled protein.

**Figure 13:**
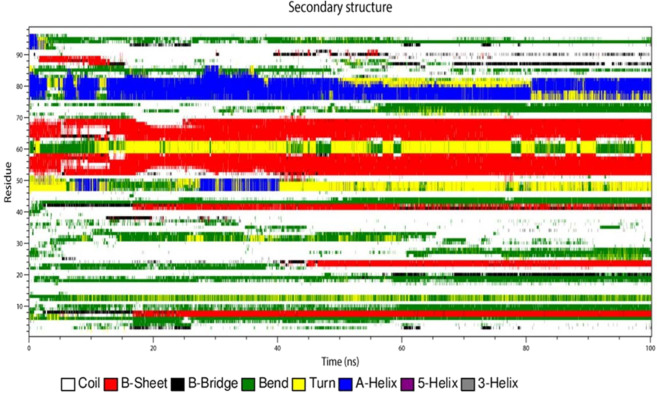
Conformational evolution of secondary structure elements of HPV E7 oncoprotein in complex with the Compound ZINC49069570.

**Figure 14:**
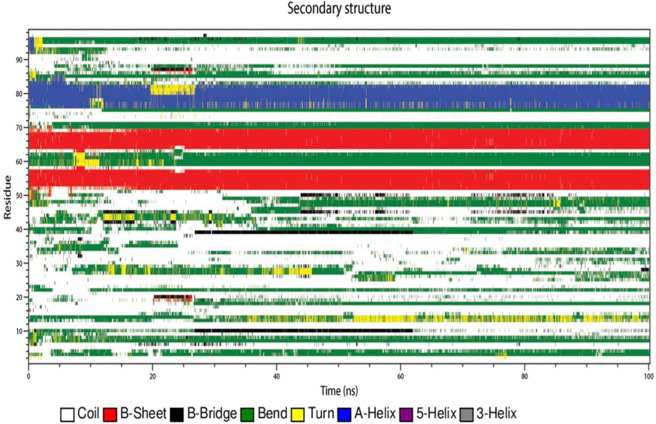
Conformational evolution of secondary structure elements of HPV E7 oncoprotein in complex with the Compound EGCG.

The secondary structure analysis of the modeled structure, best identified small molecule ZINC49069570 and EGCG was performed with the help of DSSP implemented in GROMACS which is depicted in the Figs. 12, 13 and 14 respectively. The secondary structure analysis of the modeled structure represented in Fig. 12 reveals that the residue 4 and 5 possess the conformation of bend till 30 ns simulation which is lost after the time period. The residues 8, 9 and 10 possess the conformation of turn which is transformed into the bend conformation after 40 ns simulation time. The residues 28–32 possess beta sheet conformation throughout the simulation period but also possess slight deviations between 10–20 ns of B-bridge conformation. The residues 38–44 obtained conformational changes of B-sheet and B-bridge till 20 ns simulation but later those conformations of B-sheet is maintained throughout the simulation. It also states that the residues 50–58, 64–70 in the ordered region of the modeled protein possess the B-sheet conformation which has not been lost during the simulation period. In the similar manner the residues 76–84 also possess the A-helix conformation which has been maintained throughout the simulation. The residues 85–88 containing the bend conformations has been maintained throughout the simulation with slight conformational changes. The residues 90–92 owns the bend conformation which is lost after the 40 ns time period and different conformations have been formed shows that they are not stable which is very evident with the Fig. 12. The Fig. 13 shows the secondary structure analysis of the best identified small molecule compound ZINC49069570 and it is observed that the residues 7, 8 shows the bend conformation which is maintained throughout the simulation. The residues 9, 10 shows B-bridge till 15 ns simulation period but later the B-sheet conformation has been sustained throughout the simulation. The residues 19, 20 shows bend conformation but after 60 ns the residue 20 obtained the B-bridge conformation but there was some loss of these conformations and loop conformation developed during the simulation. The residues 22–24 possess bend conformation till 35 ns and loses the conformation but after 40 ns simulation the B-sheet conformation is maintained till 100 ns simulation. These residues belong to the CR1 region and are represented as the disordered region. The residues 42–44 possess bridge conformation which is lost around the 15 ns simulation and the B-sheet conformation has been maintained throughout the simulation. The residues 48–52 contains the turns, A-helix and bend conformations throughout the simulation which is lost at one point and regained at some other point.

The residues 52–59 and 64–70 possess B-sheet conformation which is maintained throughout the simulation indicating that the identified compound does not affect the conformations of the protein. The residues 60–63 is observed to have bend and turn conformations alternatively throughout the simulation period in which turn conformation is observed more. The residues 70–76 possess conformational changes and after 55 ns the residues obtained the bend conformation till 100 ns simulation. The residues 76–82 observed to be A-helix conformations till 50 ns simulation and further have slight deviations with the 80 ns simulation and continued to be helix conformation throughout the simulation. The bend conformation visible at the residues 94–98 is maintained throughout the simulation depicting that the compound can be a potent inhibitor since the conformational changes and the domination of compounds are observed. The secondary structure analysis of the compound EGCG with the protein is observed on Fig. 14 which portrays that the residues 52–58 and 64–69 only possess B-sheet conformation and no other residues were observed for this conformation. On the whole, the complex with EGCG possess bend, B-sheet, B-bridge and A-helix conformations which shows that the best identified small molecule compound is better in comparison with the EGCG molecule. The best identified small molecule compound when bound to the HPV 16 E7 protein the disordered region containing the LXCXE motif obtained the B-sheet conformation which is very much essential for the drug discovery process since the LXCXE motif is the important sequence that binds to retinoblastoma and suppress its function.

## Discussion

The infection caused by human papillomavirus is the common sexually transmitted infection occuring mostly in women and estimated to be around 75–80% in sexually active women. Human papillomavirus infection in human develops a critical role in the common dermatologic, sexually transmitted diseases and some of the frequently occurring cancers worldwide. These infections are determined impulsively by the host immune system and in very rare cases it develops into cervical cancers when the infection persists^12^. It is surmised that for every 1 million women infected, about 100,000 will extends to precancerous modifications in their cervical tissue^10^. Infection of the stratified squamous epithelia presents in the basal cells by the virus develops replication in the suprabasal layers. Replication enzymes were not present in HPV and consequently induce entry of normally quiescent suprabasal cells to S-Phase for admittance to the host replication machinery^25^. This contribution leads to the pathogenic manifestations of the virus reigning from warts to the invasive carcinomas^15^. Numerous serotypes of HPV are present and termed as high and low risk genotypes responsible for the development of warts and cancers. Around 90% of the cancers are caused by the high risk genotypes HPV 16 and HPV18. In specific two oncoproteins namely, E6 and E7 contribute to the transformation of infected cells and the viral replication occurs in the discriminated section of epithelium where the cells have departed from the cell cycle^25^. The inhibition of the virus entry into the host cells is found to be promising and successful target in the current scenario. Disrupting the function of cellular factors indispensable for the control of cell division and proliferation is the vital role of E6 and E7 oncoprotein. However, the tumor cells which encompass E6 and E7 oncoprotein possess several transformations in the genome of the cell by distressing the cell cycle regulatory process of Retinoblastoma (pRB), p53 and tumor suppressor proteins^30^. Although E6 and E7 confer crucial transforming activities of human papillomavirus, E5 boosts the function of them which contributes towards the tumor progression^16^. Hence, we possess interest in all the three proteins. But, E7 plays a significant role in transforming activity including disruption of normal epithelial differentiation and proliferation allowing viral replication and carcinogenic transformation^14^. The vaccines Gardasil (Merck) and Cervarix (GlaxoSmithKline) for HPV infections offer anticipatory care for millions of uninfected adolescent people. However, the cost of these vaccines are not affordable by every citizens and are designed to treat only who has already infected with the human papillomavirus infection^30^. The short comings of the HPV E7 inhibitor have prompted the exploration of more natural molecules and this provoke us to implement the use of EGCG which is found to inhibit the oncogenic property of the HPV 16 E7^32,47^. Hence, it was clearly evident that targeting the HPV 16 E7 could serve as important inhibitory site for halting the entry and viral host fusion. The E7 protein shows diverse structural behaviour with intrinsically disordered N-terminal and ordered c-terminal region. Individually the terminals shows momentous role in the malignant transformation.

The work presented here has been performed with the base of our previous data and shows that the identification of inhibitors from the natural small molecule library with the help of reference molecule is strengthened by the use of various *in silico* approaches. With the identification of molecules analogue to EGCG from the natural small molecule library, the docking studies reveals that the inhibition of HPV E7 with EGCG is not so appreciable when compared to the identified molecules. Around eleven molecules have been observed to be the best analogue compounds inveterate over the assistance of shape similarity score. The significant site of HPV is the CR3 region and reports says that this site contains patch1 sequence of amino acids required for pRB binding^39^. Since this regions has its contribution in the displacement of E2F from pRB which leads to the transformation, this has been concentrated for the study. Hence, the binding site of HPV 16 E7 within the CR3 region is made ready for the use of docking strategy with the identified small molecules and this shows that the compound ZINC49069570 and ZINC49115270 possess better docking score and binding energy in comparison with the EGCG and other identified molecules. It is also observed that each compound binds to E7 oncoprotein at the flexible loop of both terminal. It is very evident that EGCG binds and hijacks the flexibility of the protein through binding along with the N and C terminal. The identified molecules and the reference compound EGCG were optimized to identify the atoms responsible for the interaction with the receptor. The electronic structure calculations were carried out to signifies the electron transfer which contributes extremely towards the molecular interactions. The electrostatic potential surface of the compounds ZINC49069570, ZINC49115270 and EGCG represented in Fig. 6 states that the compounds possess attractive potential in the atoms that develops interaction with receptor. The analyses provided better insights that the interaction is on the attractive region and mostly on the moderate region which plays noteworthy role in the interaction and authorize the consequence of the compounds inhibition.

The electron transfers of the compounds depicts that there is chance of accepting and donating electrons from one region of the compound to the other region of the compound which provides strong interaction with the protein. It has been clearly evident with the results that these compounds are reactive stating the impact on the effect of inhibition against the oncoprotein. When the simulation studies have been witnessed it was apparent that the beta structure changes its conformation into the partial helix. The conformational changes is also supported by the free thread followed along with the terminal region. The post docking binding free energy calculation states that the compound ZINC49069570 and ZINC49115270 possess highest binding free energy depicting the strength of compound inhibition. The molecular dynamics simulation studies, have demonstrated that the compound ZINC49069570 and ZINC49115270 shows its involvement in the inhibition of the oncoprotein through its conformational stability and the changes. The simulation has been carried out thrice for the period of 100 ns in order to validate the results of simulation for high significant belief on the theoretical studies. And fortunately from the results it is understood clearly that only a slight deviation and the difference of 0.01 Å has been witnessed stating that there is no significant changes in the various simulation. These studies also states that the simulation is statistically valid and the difference observed will not develop much problems in the data’s obtained and the results inferred. Interestingly, the mean RMSD states that the compound ZINC49069570 shows higher stability when compared to the apoprotein and the other molecules including EGCG. The conformational changes are also observed better with the compounds binding and witnessed to be more than the EGCG molecule. Our simulations also indicates a significant maintenance of the hydrogen bonds in the complex formation. In order to further access the motion of the protein with its complexes, the principle component analysis has been performed which portrays that the compound ZINC49069570 have collective motions when compared with the EGCG. The compound ZINC49069570 possess a minimal conformational phase space depicting that the compound can be better inhibitor of the protein. We have also observed the secondary structure elements of the protein by analyzing the trajectory of molecular dynamics simulation and it states that the compound ZINC49069570 dominates the protein and acquired the conformational changes depicting the stability of the protein in contact with the ligand when compared with the reference molecule. The post dynamics binding free energy calculation associates the importance of the compound ZINC49069570 even after the 100 ns simulation. Insights gained from the study could assist the identification of novel inhibitors analogue to EGCG that could exploit the conformation of the protein which in turn inhibits the oncogenic property of the protein E7. These results can be further employed for the wet lab examination and could deliver suggestion for the inhibition of HPV E7 oncoprotein in association with the cervical cancer.

## Supplementary information

Supplementary Information.
