# Supplementary material for: Structural dynamic studies on identification of EGCG analogues for the inhibition of Human Papillomavirus E7
Source: Sci Rep. 2020 May 26;10:8661. doi: 10.1038/s41598-020-65446-7 (PMC7250877; doi:10.1038/s41598-020-65446-7)
Supplement: Supplementary file 1 — Supplementary Information. [file 41598_2020_65446_MOESM1_ESM.docx]

**Structural dynamic studies on identification of EGCG analogues for the inhibition of Human Papillomavirus E7**

**Murali Aarthy, Umesh Panwar and Sanjeev Kumar Singh***

**Computer Aided Drug Design and Molecular Modeling Lab, Department of Bioinformatics, Alagappa University, Karaikudi - 630004**

*E-mail: [skysanjeev@gmail.com](mailto:skysanjeev@gmail.com)

Running Title: Ligand based screening and molecular simulation studies on targeting HPV E7 oncoprotein.

**Supplementary Figure 1:**


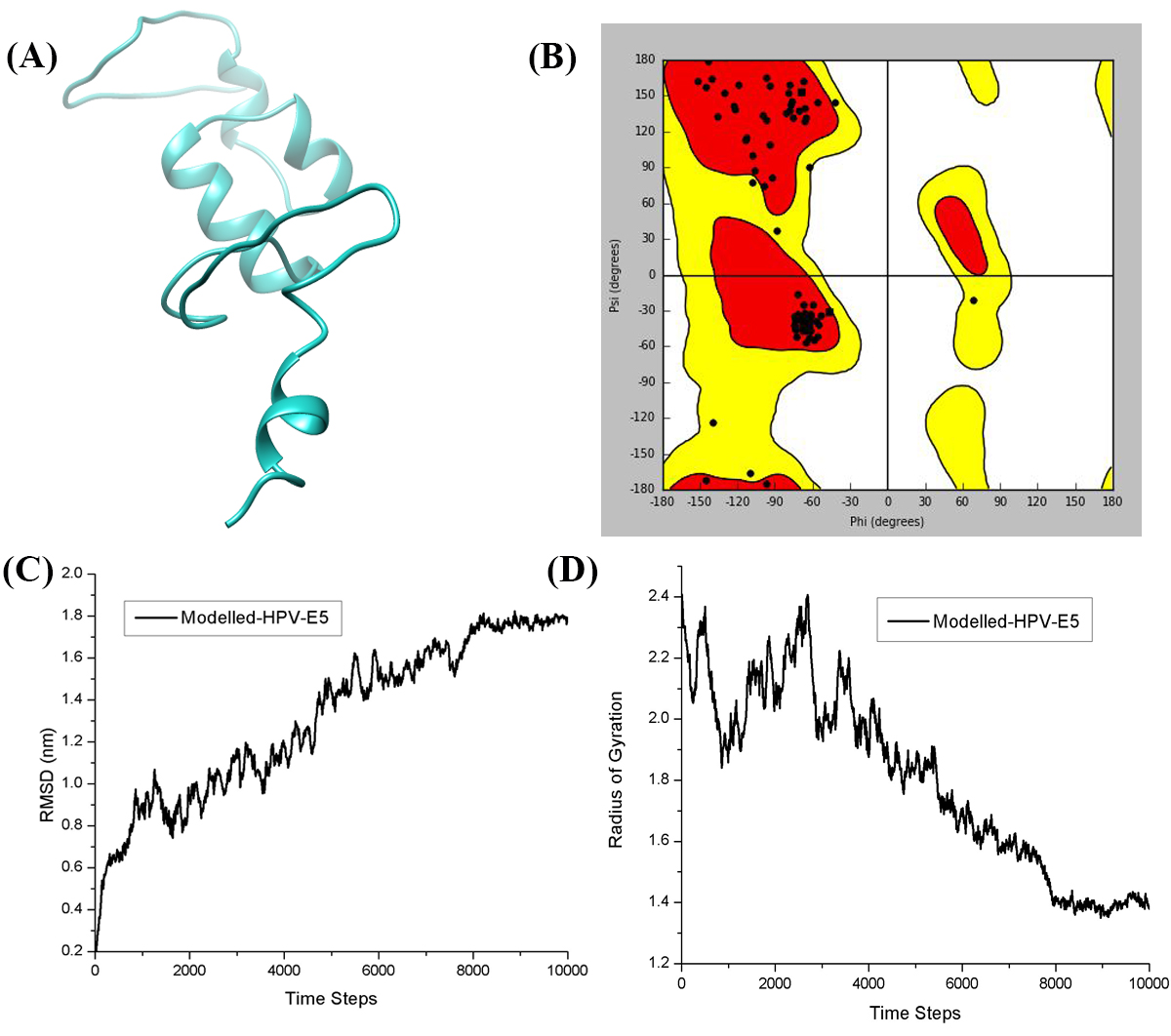


**Sup. Figure 1: (a) represents the homology modeled structure of HPV E5 performed through Modeller with the template PDB ID: 4L9P. (b) Represents the ramachandran plot validation of the E5 protein stating that the proteins possess reliability in terms of φ and ψ angles. The plot shows that there are no residues in the disallowed region and 96 percent residues fall in the most favored region.**

The molecular dynamics results in C and D of Supplementary Fig 1 states that the E5 protein attains stability after 8ns and maintained till 10ns. The low energy conformation has been obtained after 10ns simulation and used further for molecular docking studies.

**
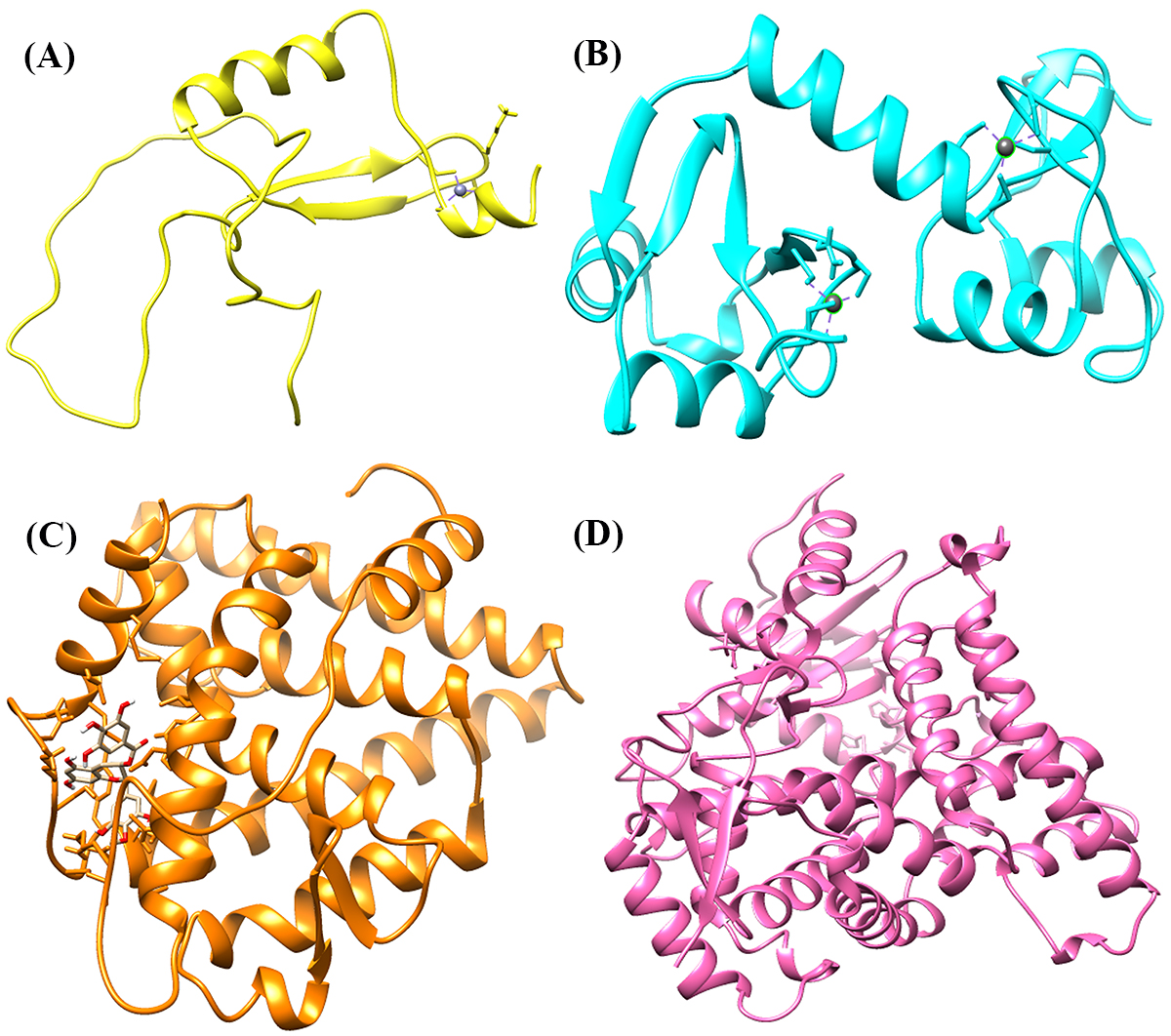
**

**Sup. Figure 2: (A) represents the homology modeled structure of HPV E7 performed through Modeller. (B) Represents the three dimensional structure of HPV E6. (C) Represents the three dimensional structure of Aromatase used in the study. (D) Represents the three dimensional structure of ER alpha used in the study.**

**Table 1: XYZ co-ordinates for the binding site of the oncoproteins E5, E6,aromatase & ERα observed for docking with EGCG and its analogues**

| **S.No** | **Protein name** | **X** | **Y** | **Z** |
| --- | --- | --- | --- | --- |
|  | E7 | 69.97 | 80.16 | 10.5 |
|  | E5 | 35.96 | 1.08 | -17.12 |
|  | E6 | -54.18 | -6.86 | -11.13 |
|  | Aromatase | 85.65 | 54.17 | 45.84 |
|  | ERα | 14.12 | 3.65 | 36.48 |

The protein E5 and E6 does not have any co-crystallized ligand molecules hence, Site Map module implemented in Schrodinger is used whereas for the receptor aromatase and ERα co-crystallized ligand is present and the ligand were picked manually to predict the binding site of the receptors in order to dock the EGCG and its analogues.

**Table 2: Docking scores for E5, E6, aromatase & ERα with EGCG and its analogues**

| **S.No** | **Compound Name** | **Docking Score** | | | | **Glide Energy** | | | |
| --- | --- | --- | --- | --- | --- | --- | --- | --- | --- |
|  |  | **E5** | **E6** | **Aromatase** | **ERα** | **E5** | **E6** | **Aromatase** | **ERα** |
|  | ZINC49069570 | -4.713 | -6.639 | -11.493 | -13.680 | -40.675 | -44.525 | -59.703 | -56.393 |
|  | ZINC49115270 | -5.859 | -7.010 | -10.467 | -6.582 | -33.068 | -48.323 | -60.751 | -50.635 |
|  | ZINC14436185 | -3.890 | -6.974 | -10.828 | -9.230 | -33.876 | -45.183 | -61.386 | -51.217 |
|  | ZINC14642643 | -6.050 | -5.652 | -10.498 | -9.100 | -36.895 | -44.572 | -58.413 | -55.783 |
|  | NCI-714028 | -6.658 | -5.205 | -10.441 | -14.298 | -48.074 | -46.947 | -80.904 | -59.251 |
|  | ZINC03978503 | 5.860 | -5.188 | -10.439 | -14.209 | -38.994 | -43.463 | -60.447 | -57.839 |
|  | ZINC06040160 | -5.124 | -4.973 | -11.125 | -13.786 | -36.230 | -44.398 | -62.638 | -57.652 |
|  | ZINC84428482 | -4.904 | -6.271 | -11.559 | -8.130 | -35.611 | -44.803 | -60.919 | -42.244 |
|  | ZINC03870412 | -6.430 | -5.935 | -12.068 | -9.066 | -35.634 | -45.563 | -61.375 | -38.947 |
|  | NCI-636594 | -5.497 | -5.098 | -10.295 | -14.348 | -44.240 | -43.175 | -83.251 | -55.732 |
|  | ZINC85648240 | -4.649 | -4.812 | -10.804 | -8.624 | -29.886 | -44.760 | -85.343 | -53.328 |
|  | EGCG | -6.925 | -5.689 | -10.842 | -9.621 | -35.790 | -46.507 | -62.934 | -50.061 |

The docking scores of the oncoproteins E5, E6, Aromatase and ERα has shown better energies during the glide XP docking but when the interaction profiles of these targets have been analysed it shows less interactions when compared with E7. Also, E7 plays a significant role in transforming activity including disruption of normal epithelial differentiation and proliferation allowing viral replication and carcinogenic transformation^14^ we have concentrated on E7 activities and its inhibition. The interaction images of the analogues and EGCG with E5, E6, aromatase and ERα have been represented in Supplementary Figure 2, 3, 4, & 5. When the interaction profiles were observed, it is clearly evident that strong interaction profiles were observed in E5, E6, Aromatase, ERα, since all the best hit compounds were analogues to each other.


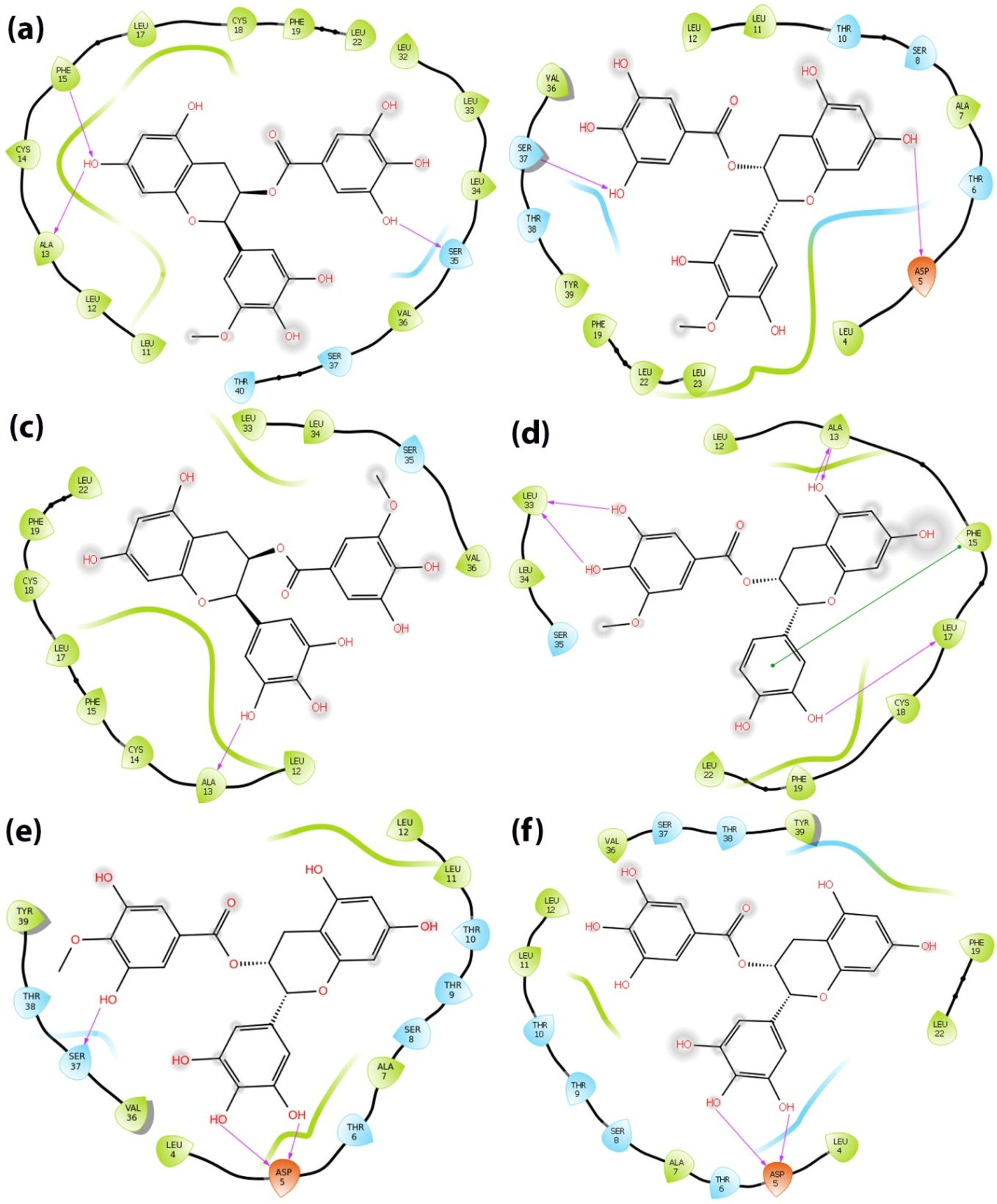


**Sup. Figure 3: Interaction profile of the best 5 compounds along with the EGCG observed for the inhibition with E5 Oncoprotein.**


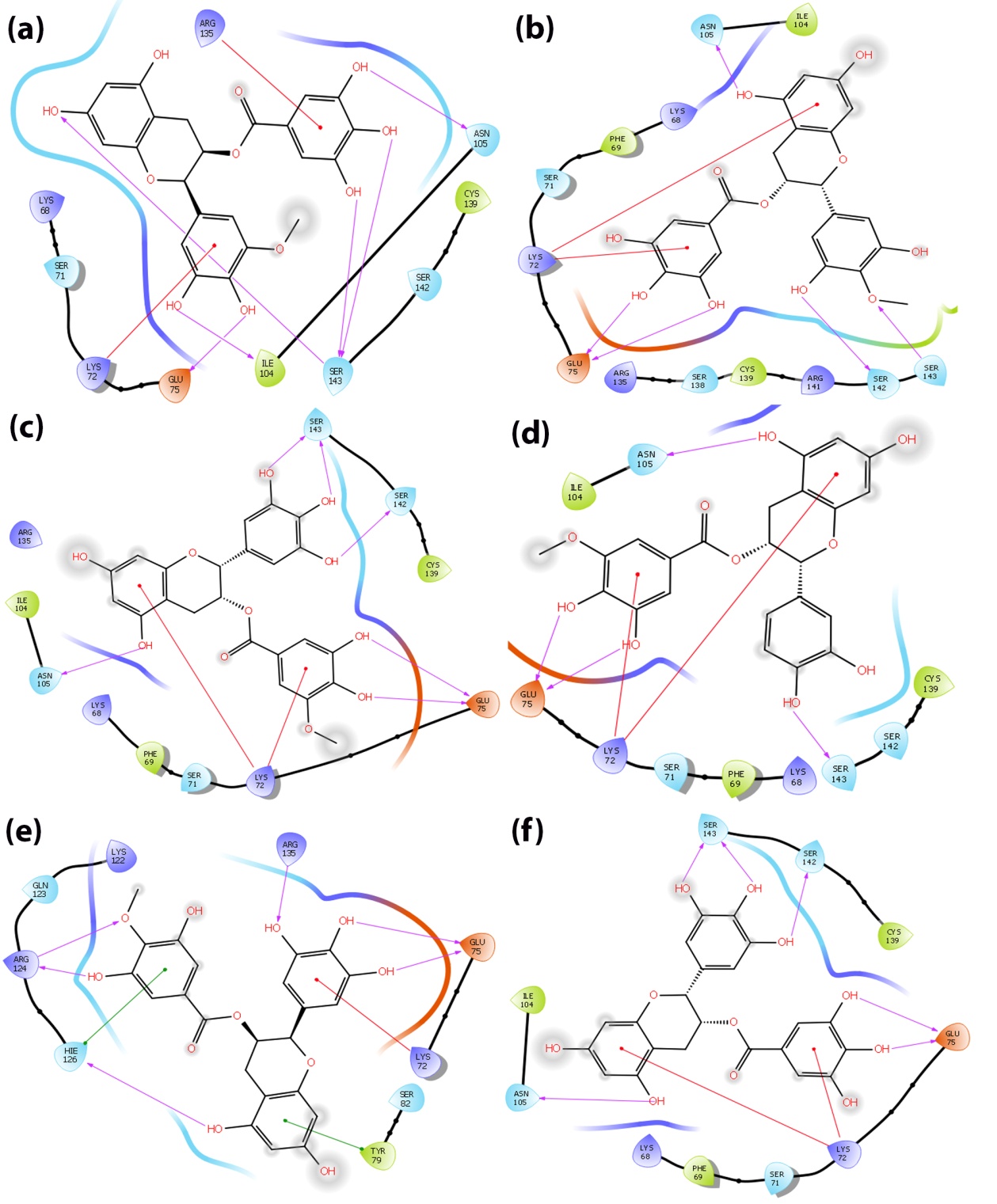


**Sup. Figure 4: Interaction profile of the best 5 compounds along with the EGCG observed for the inhibition with E6 oncoprotein.**

**
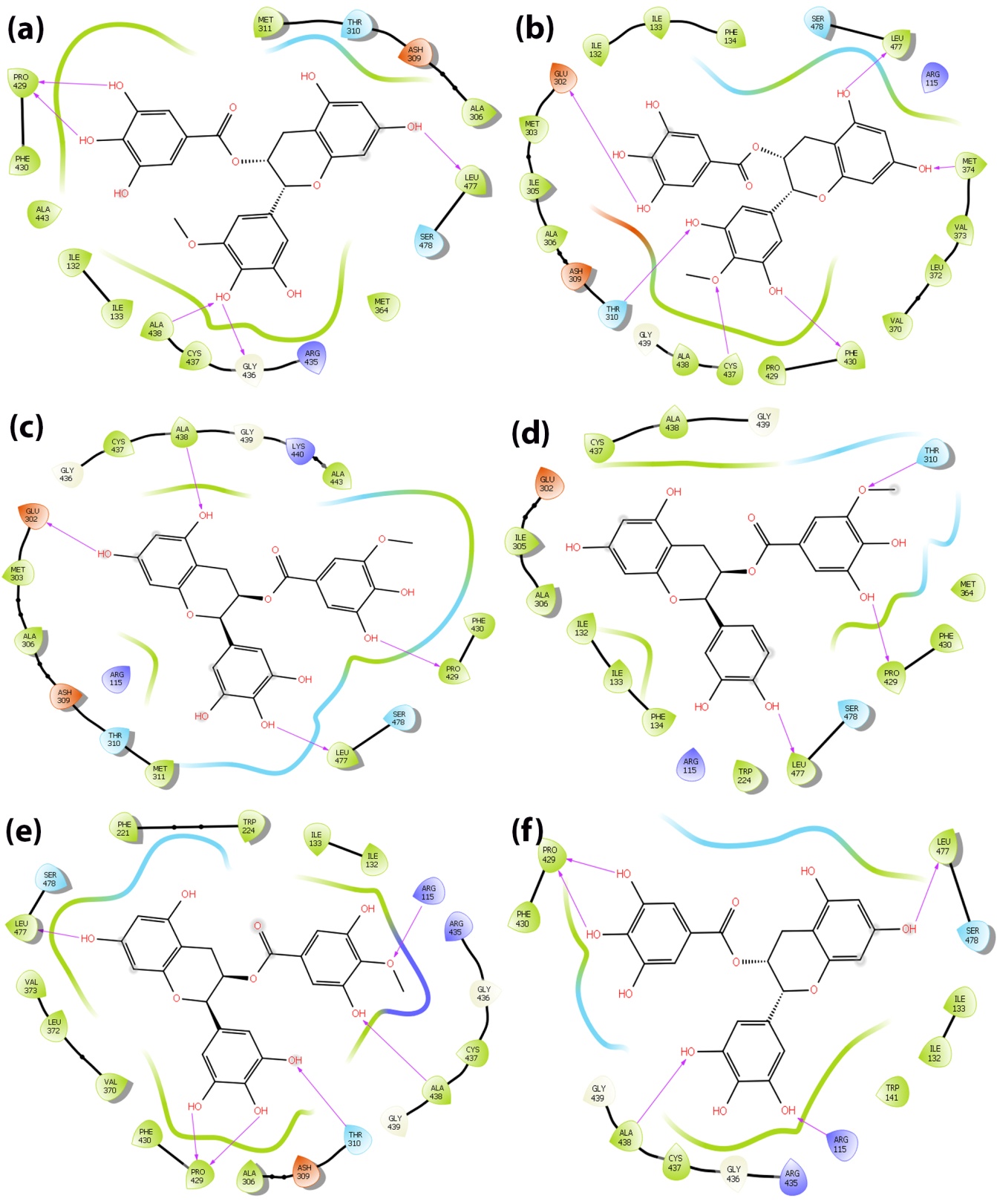
**

**Sup. Figure 5 Interaction profile of the best 5 compounds along with the EGCG observed for the inhibition with aromatase.**

**
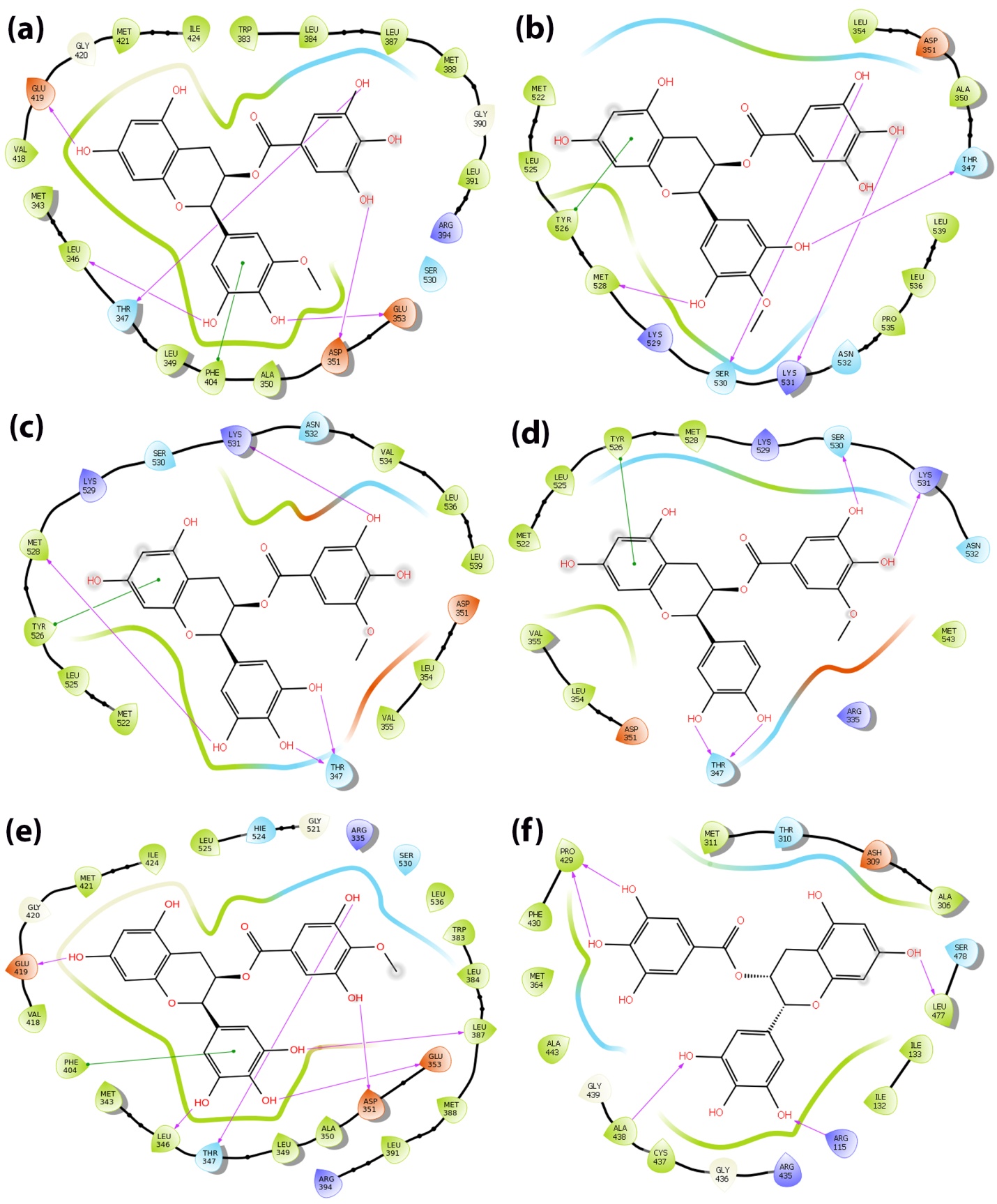
**

**Sup. Figure 6: Interaction profile of the best 5 compounds along with the EGCG observed for the inhibition with ERα.**

**
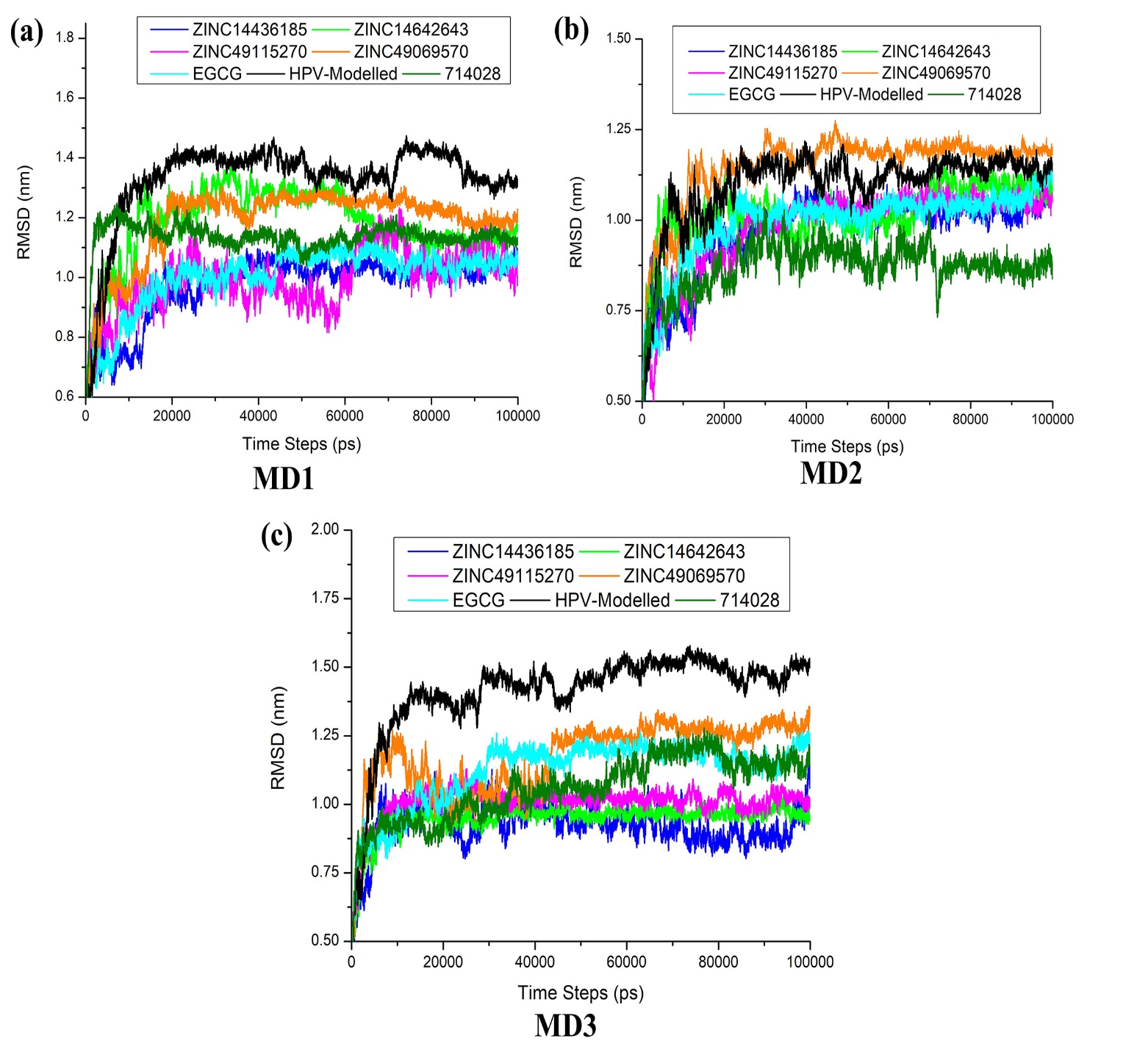
**

**Sup. Figure 7: The RMSD analysis from simulation results for the three simulation MD1, MD2 and MD3 for the apoprtein and complexes for the period 100ns (each 100ns). (a) RMSD results obtained during first simulation run, (b) RMSD results obtained during second simulation run, (c) RMSD results obtained during third simulation run.**


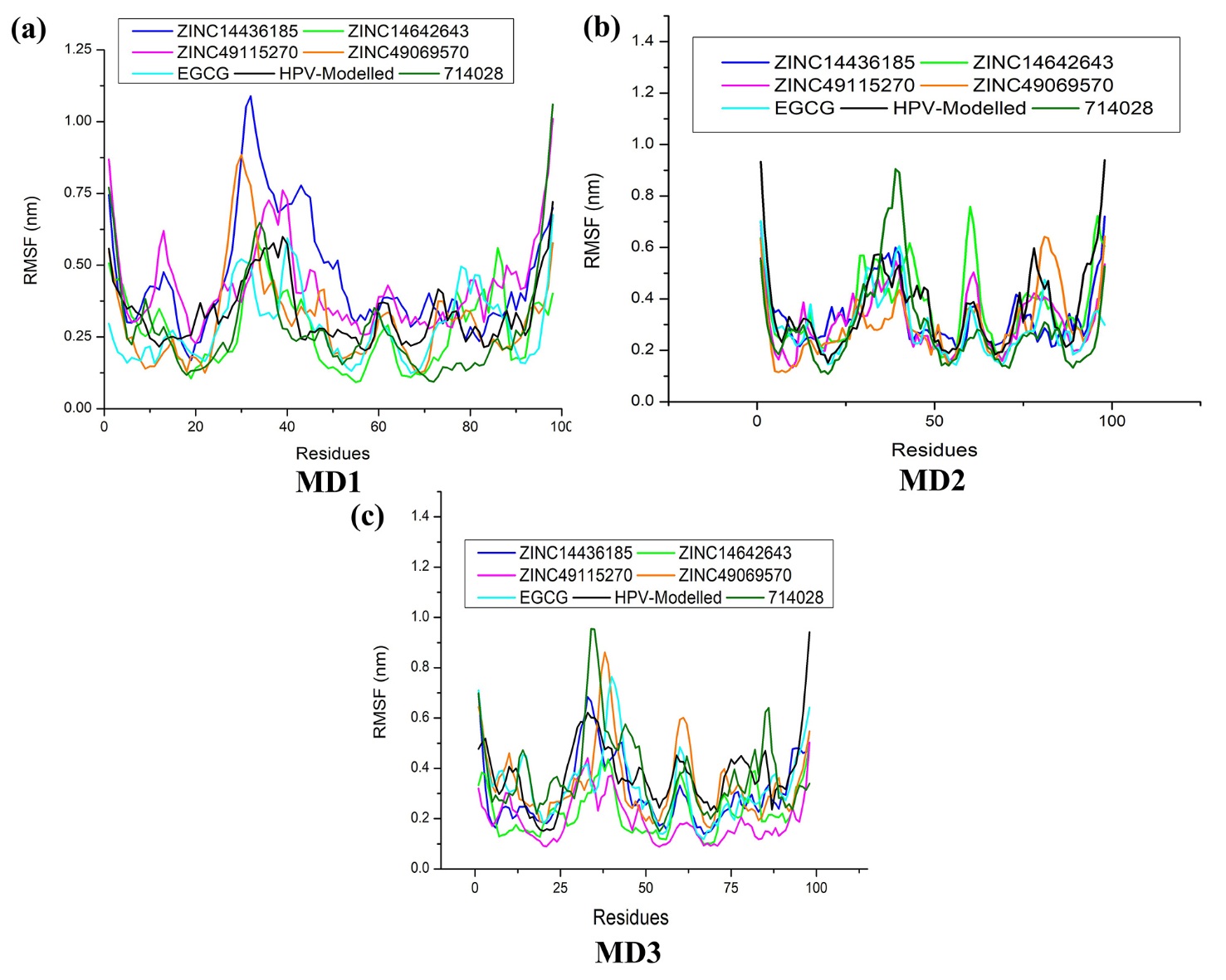


**Sup. Figure 8: The RMSF analysis from simulation results for the three simulation MD1, MD2 and MD3 for the apoprtein and complexes for the period 100ns (each 100ns). (a) RMSF results obtained during first simulation run, (b) RMSF results obtained during second simulation run, (c) RMSD results obtained during third simulation run.**

**
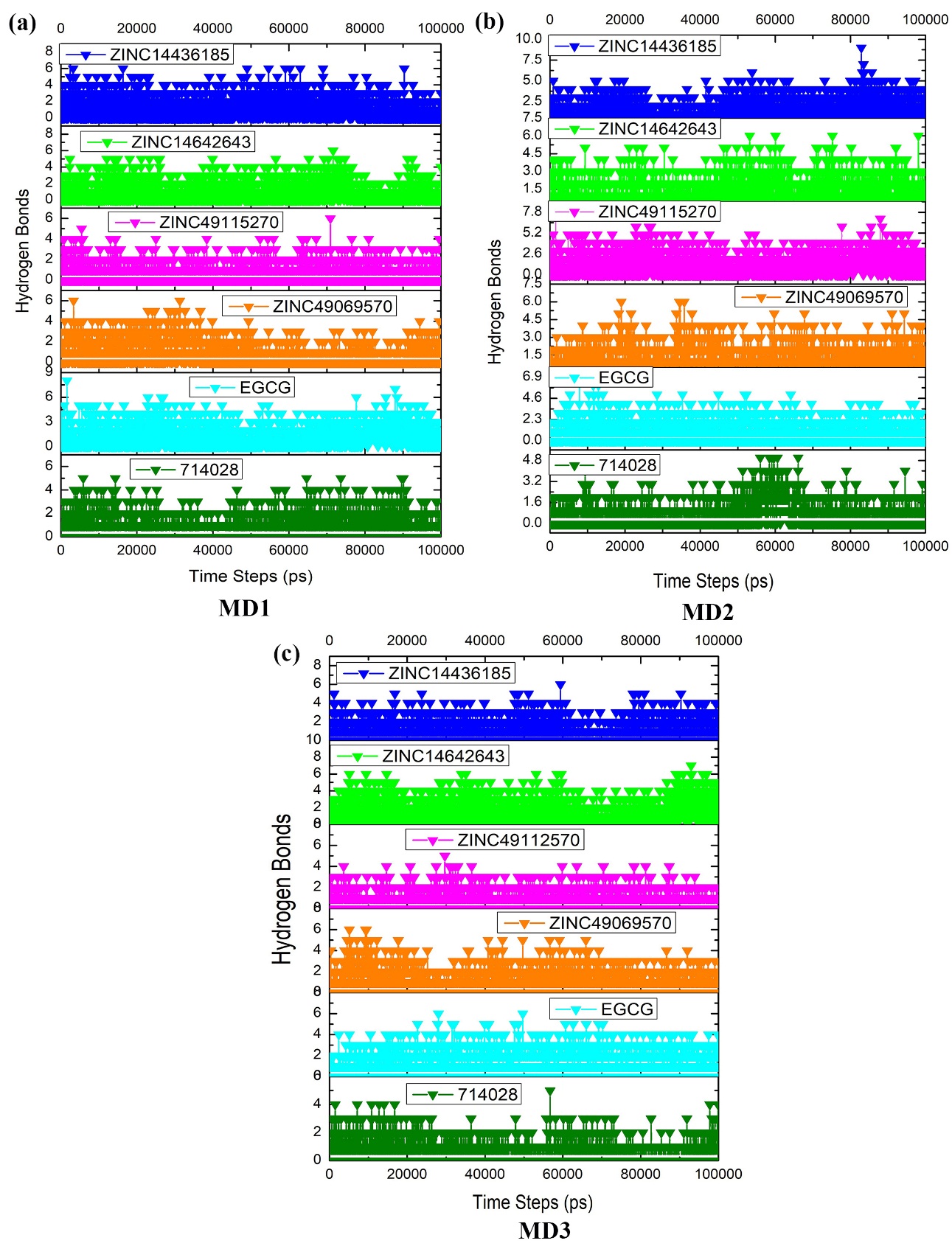
**

**Sup. Figure 9: The H-bond analysis from simulation results for the three simulation MD1, MD2 and MD3 for the apoprtein and complexes for the period 100ns (each 100ns). (a) H-Bond results obtained during first simulation run, (b) H-Bond results obtained during second simulation run, (c) RMSD results obtained during third simulation run.**
